# One-Pot Synthesis of 3,4-Dihydrocoumarins via C-H Oxidation/Conjugate Addition/Cyclization Cascade Reaction

**DOI:** 10.3390/molecules28196853

**Published:** 2023-09-28

**Authors:** Dae Young Kim

**Affiliations:** Department of Chemistry, Soonchunhyang University, Asan 31538, Chungnam, Republic of Korea; dyoung@sch.ac.kr

**Keywords:** organocatalysis, one-pot reaction, *ortho*-quinone methides, dihydrocoumarins, oxazolones

## Abstract

The 3,4-dihydrocoumarin derivatives were obtained from 2-alkyl phenols and oxazolones via C–H oxidation and cyclization cascade in the presence of silver oxide (Ag_2_O) and *p*-toluenesulfonic acid as a Brønsted acid catalyst. This approach provides a one-pot strategy to synthesize the multisubstituted 3,4-dihydrocoumarins with moderate to high yields (64–81%) and excellent diastereoselectivity (>20:1).

## 1. Introduction

The dihydrocoumarin core is present as a characteristic structural motif to possess a broad range of biological activities [1,2]. Particularly, 3,4-dihydrocoumarins have attracted considerable attention due to their various pharmacological properties, such as antiviral, anti-inflammatory, and anticancer effects [3,4]. Therefore, various novel methods for the synthesis of 3,4-dihydrocoumarins have been developed [5,6,7,8,9,10,11]. The most general protocol for the synthesis of 3,4-dihydrocoumarins is the [4 + 2] cycloaddition of *ortho*-quinone methides (*o*-QMs) [12,13,14,15,16]. *ortho*-Quinone methides are synthetic intermediates widely used in organic synthesis [17,18]. A variety of useful reactions of *ortho*-quinone methides with several dipoles have been reported using transition metal catalysts [19,20] or organic catalysts [21,22]. Oxazolone derivatives have been recognized as well-known precursors of α-amino acids, are versatile building blocks in organic synthesis, and are often used in the synthesis of α,α-disubstituted α-amino acids [23].

**Scheme 1:**
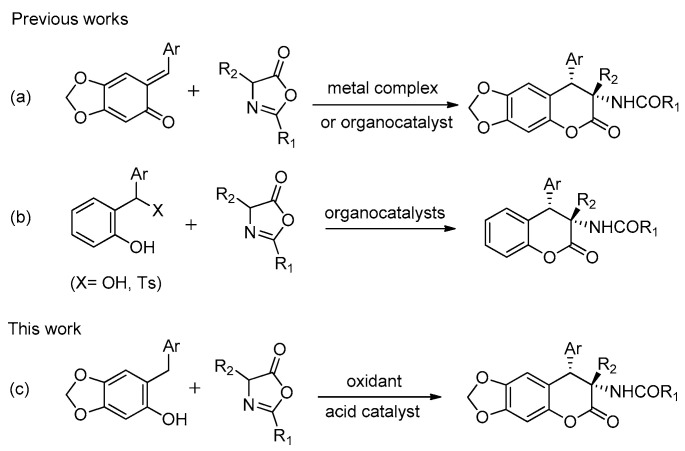
Methods for the asymmetric synthesis of dihydrocoumarins.

Many studies have been reported to synthesize 6-membered oxacyclic compounds through [4 + 2] cycloaddition by reacting *o*-QM with various 1,2-dipoles [24,25]. Recently, the synthesis of 3,4-dihydrocoumarin derivatives by cycloaddition reaction between *o*-QM and oxazolones using a metal complex or hydrogen bonding organocatalyst has been reported (Scheme 1a) [26,27]. Additionally, the [4 + 2] ring addition of *o*-QM precursor and oxazolone using an organic catalyst has been reported (Scheme 1b) [28,29]. Despite this progress, the development of new and efficient synthetic methods for the synthesis of 3,4-dihydrocoumarin is still highly desirable. Cascade cyclization reactions have attracted much attention in the past few decades, providing practical protocols for the synthesis of heterocyclic molecules with the convenience afforded by shortening the reaction steps [29,30]. To the best of our knowledge, the single-pot synthesis of dihydrocoumarins from the in situ generation of *o*-QMs via C–H oxidation of benzyl phenol has not been reported. Therefore, we envisioned a one-pot synthesis of 3,4-dihydrocoumarin derivatives via C–H oxidation and ring closure cascade of 2-benzyl phenol with oxazolone under Brønsted acid conditions (Scheme 1c). In connection with our work on conjugate addition reactions, [31,32], we reported the Michael-type addition/ring closure sequences of *o*-QM with dipoles [33]. Herein, we describe the one-pot synthesis of 3,4-dihydrocoumarin derivatives from 2-benzyl phenol via C–H oxidation and acid-catalyzed ring closure cascade.

## 2. Results and Discussion

We commenced our study with 6-(4-methoxybenzyl)benzo[*d*][1,3]dioxol-5-ol (**1a**) and 4-benzyl-2-phenyloxazol-5(4*H*)-one (**2a**) as model substrates in the presence of oxidant and Brønsted acid catalyst, which was previously validated as the suitable catalysts for conjugate addition reactions [34,35]. 

The initial study was conducted with Ag_2_CO_3_ as an oxidant for the in situ generation of *ortho*-quinone methide intermediate via C-H oxidation. The desired 3,4-dihydrocoumarin (**3a**) was obtained in a 35% yield with 10 mol% of *p*-toluenesulfonic acid (Table 1, entry 1). To further improve the yield, organic and metallic oxidants, such as Ag_2_O, AgOAc, AgOTf, AgBF_4_, AgNO_3_, MnO_2_, Mn(OAc)_3_, Mn(acac)_3,_ K_2_S_2_O_8_, Oxone, TBHP (*tert-*butyl hydroperoxide), 2,3-dichloro-5,6-dicyano-1,4-benzoquinone (DDQ), (diacetoxyiodo)benzene, and PIFA ([bis(trifluoroacetoxy)iodo]benzene) were evaluated for their potential impact on this reaction. Silver oxide (Ag_2_O) proved to be a suitable oxidant with regard to yield (Table 1, entry 2). Ag_2_O has the advantage of excellent thermal stability and is commercially available in various forms (e.g., powder, pellets, flakes, etc.). Next, we examined the evaluation of the efficiency of selected Brønsted acids, including methanesulfonic acid, 2,4-dinitroebenzene sulfonic acid, acetic acid, trifluoroacetic acid, and diphenyl phosphate (DPP) (Table 1, entries 2 and 16–20). Diphenyl phosphate was the optimal catalyst for the oxidative cyclization cascade reaction (81% yield, Table 1, entry 20). Then, other common solvents such as methylene chloride, 1,2-dichloroethane (DCE), ethyl acetate, diethyl ether, tetrahydrofuran (THF), dioxane, benzene, toluene, and *p*-xylene were tested in the reaction (Table 1, entries 2 and 21–29). Chloroform was confirmed as the optimum solvent in terms of yield (Table 1, entry 20).

**Scheme 2:**
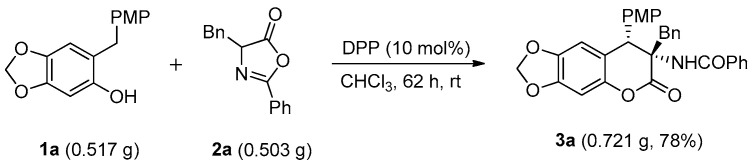
The gram-scale synthesis of dihydrocoumarin **3a**.

After establishing the optimized conditions, the substrate range of oxazolone derivative **2** was investigated. In reactions with various oxazolone derivatives (**2**), the corresponding 3,4-dihydrocoumarin derivatives **3** were obtained in reasonable yields (Scheme 2). The reactions of 5-(4*H*)oxazolones (**2**) with either electron-donating (4-OMe, 4-Me, 3-Me, and 3,5-di-Me_2_) or electron-withdrawing (4-CF_3_, 4-Cl, and 2-Cl) substituents on the phenyl ring in the C2 position yielded the corresponding 3,4-dihydrocoumarins (**3b**–**3h**) in high yields (Table 2, 68–77% yields). In addition, when there are p-halo-substituted (F, Cl) benzyl and (methylthio)ethyl groups instead of the benzyl group in the C4 position of 5-(4*H*)oxazolone (**2**), the corresponding 3,4-dihydrocoumarins (**3i**–**3k**) were processed in reasonable yields and (Table 2, 64–72 yields). In all cases, the diastereoselectivity of the products (**3**) was excellent (over 20:1). The relative configuration of the indicated diastereomer was determined by ^1^H-NMR analysis compared with reported works [26,27].

**Scheme 3:**
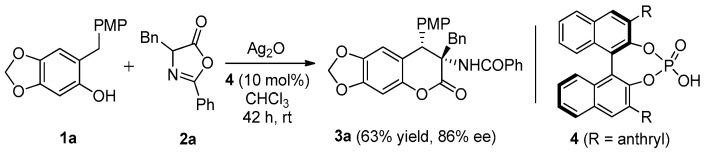
Asymmetric synthesis of 3,4-dihydrocoumarins **3a**.

In order to demonstrate the synthetic utility of this transformation, the gram-scale synthesis of 3,4-dihydrocoumarin (**3a**) was conducted. As shown in Scheme 2, when 2-benzyl phenol (**1a**) was treated with 4-benzyl-2-phenyloxazol-5(4*H*)-one (**2a**) under optimal reaction conditions, the reaction proceeded well to afford the chiral 3,4-dihydrocoumarin (**3a**) with a 78% yield. To achieve the asymmetric version of this reaction, the reaction was conducted with a chiral phosphoric acid catalyst (**4**) instead of diphenyl phosphate under standard reaction conditions (Scheme 3). The chiral 3,4-dihydrocoumarin (**3a**) obtained a 63% yield with 86% enantioselectivity. It is necessary to further investigate the structure of the catalyst and optimize reaction conditions suitable for asymmetric reactions, and further research is still being carried out in our laboratory.

**Figure 1:**
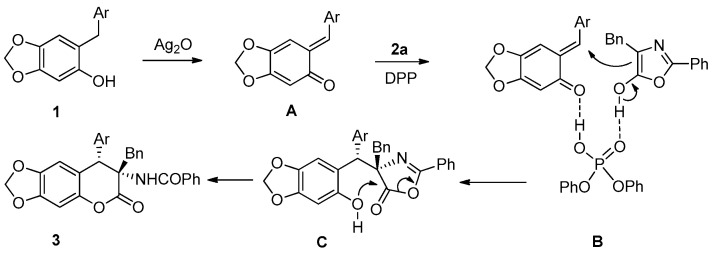
Proposed reaction mechanism.

Based on the experimental results, a plausible reaction pathway and activation mode were proposed (Figure 1). The *ortho*-quinone methide intermediate A was generated in situ from benzylphenol (**1**) in the presence of Ag_2_O as an oxidant. Then, diphenyl phosphate simultaneously generated two hydrogen bonds with both the *ortho*-quinone methide intermediate and enol-form of oxazolone (**2a**). The subsequent conjugate addition of the C4 of oxazolone enolate to *ortho*-quinone methide leads to the Michal-type adduct (**C**). The intermediate (**C**) would undergo an annulation (lactonization) reaction with a concomitant opening of the azlactone ring to produce cyclic α-acylaminolactone (**3**). 

## 3. Materials and Methods

All chemicals were purchased from commercial suppliers and used without further purification unless otherwise specified. Solvents for extractions and chromatography were of technical grade and were distilled prior to use. Extracts were dried over technical-grade anhydrous Na_2_SO_4_. Anhydrous solvents were deoxygenated by sparging with N_2_ and dried by passing through activated alumina columns of a Pure Solv solvent purification system (Innovative Technology). Reactions were monitored by analytical thin-layer chromatography (TLC) using silica gel 60 F_254_ pre-coated glass plates (0.25 mm thickness) and visualized using UV light (254 nm and 365 nm), I_2_, *p*-anisaldehyde, ninhydrin, and phosphomolybdic acid solution as an indicator. Flash chromatography was carried out on E. Merck silica gel (230–400 mesh). ^1^H NMR, ^13^C NMR, and ^19^F NMR spectra were recorded at 400 MHz, 100 MHz, and 376 MHz, respectively, on a Jeol ECS 400 MHz NMR spectrometer. Chemicals shift values (δ) are reported in parts per million and referenced in relation to the following standards: Me_4_Si as the internal references for the ^1^H- and ^13^C-NMR signals in chloroform and PhCF_3_ as the external references for the ^19^F-NMR signal. The peak information is described as s = singlet, d = doublet, t = triplet, q = quartet, and m = multiplet. Mass spectra (MS-EI, 70 eV) were conducted on a GC-MS Shimadzu QP2010. High-resolution mass spectra were measured on a Jeol HX110/110A using the electrospray ionization technique. The enantiomeric excesses (EEs) were determined by HPLC. HPLC analysis was performed on a Shimadzu prominence 20, measured at 254 nm using the indicated chiral column. Optical rotations were measured on a JASCO-DIP-1000 digital polarimeter with a sodium lamp. Infrared spectra were recorded on a Thermo Fisher Scientific Nicolet iS5 FT-IR spectrometer. The elemental analysis was carried out on a Perkin-Elmer 2400 Series II Elemental Analyzer.

*General procedure for the one-pot synthesis of 3,4-dihydrocoumarins* **3***:* To a stirred solution of 2-benzylphenol **1** (0.2 mmol), oxazolone **2** (0.2 mmol), and Ag_2_O (0.24 mmol) in chloroform (3 mL), diphenyl phosphate (0.02 mmol) was added at room temperature under an N_2_ atmosphere. The reaction mixture was stirred for 24–36 h at room temperature. After completion of the reaction (monitored by TLC), the solvent was removed under reduced pressure. The crude product was purified by flash chromatography (EtOAc/hexane = 1:5) to afford 3,4-dihydrocoumarins **3**.

*N-(7-benzyl-8-(4-methoxyphenyl)-6-oxo-7,8-dihydro-6H-[1,3]dioxolo [4,5-g]chromen-7-yl)benzamide* (**3a**), 81% yield; ^1^H NMR (400 MHz, CDCl_3_) δ 7.45–7.41 (m, 2H), 7.20–7.16 (m, 3H), 7.05–7.02 (m, 4H), 6.82 (d, *J* = 8.8 Hz, 2H), 6.80 (s, 1H), 6.69–6.68 (m, 3H), 6.64 (s, 1H), 5.99 (dd, *J* = 16.0, 1.6 Hz, 2H), 5.27 (s, 1H), 4.13 (d, *J* = 14.0 Hz, 1H), 3.67 (s, 3H), 3.13 (d, *J* = 14.1 Hz, 1H); ^13^C NMR (100 MHz, CDCl_3_) δ 168.5, 168.0, 159.0, 148.1, 144.4, 135.2, 135.0, 131.7, 130.2, 129.9, 129.0, 128.7, 128.5, 127.4, 126.8, 117.8, 114.2, 108.7, 102.1, 98.5, 77.4, 77.1, 76.8, 66.0, 55.2, 49.2, 38.2; IR: 3356, 2970, 1764, 1666, 1502, 1481, 1324, 1128 cm^−1^; HRMS (ESI): *m*/*z* calcd for C_31_H_25_NNaO_6_ [M + Na]^+^: 530.1580; found 530.1585; Elemental Analysis for C_31_H_25_NO_6_: C, 73.36; H, 4.96; N, 2.76; O, 18.91. Found: C, 73.34; H, 4.98; N, 2.75; O, 18.93.

*N-(7-benzyl-8-(4-methoxyphenyl)-6-oxo-7,8-dihydro-6H-[1,3]dioxolo [4,5-g]chromen-7-yl)-4-methoxybenzamide* (**3b**), 74% yield; ^1^H NMR (400 MHz, CDCl_3_) δ 7.46–7.40 (m, 2H), 7.23–7.16 (m, 3H), 7.05–6.98 (m, 4H), 6.52 (d, *J* = 8.8 Hz, 2H), 6.71–6.69 (m, 3H), 6.64 (s, 1H), 5.99 (dd, *J* = 16.0, 1.6 Hz, 2H), 5.28 (s, 1H), 4.35 (d, *J* = 14.0 Hz, 1H), 3.80 (s, 3H), 3.67 (s, 3H), 3.14 (d, *J* = 14.0 Hz, 1H); ^13^C NMR (100 MHz, CDCl_3_) δ 168.6, 167.4, 162.4, 159.0, 148.0, 145.3, 144.4, 135.1, 130.3, 130.0, 129.0, 128.7, 128.4, 127.4, 127.4, 117.9, 114.1, 113.8, 108.7, 102.1, 98.5, 65.9, 55.5, 55.2, 49.2, 38.2; IR: 3342, 2971, 1762, 1675, 1501, 1491, 1324, 1129 cm^−1^; HRMS (ESI): *m*/*z* calcd for C_32_H_27_NNaO_7_ [M + Na]^+^: 560.1685; found 560.1681; Elemental Analysis for C_32_H_27_NO_7_: C, 71.50; H, 5.06; N, 2.61; O, 20.83. Found: C, 71.47; H, 5.08; N, 2.60; O, 20.85.

*N-(7-benzyl-8-(4-methoxyphenyl)-6-oxo-7,8-dihydro-6H-[1,3]dioxolo [4,5-g]chromen-7-yl)-4-methylbenzamide* (**3c**), 77% yield; ^1^H NMR (400 MHz, CDCl_3_) δ 7.38 (d, *J* = 6 Hz, 2H), 7.20–7.10 (m, 3H), 7.05 (d, *J* = 8.7 Hz, 4H), 6.80 (s, 1H), 6.70 (s, 4H), 5.98 (s, 2H), 6.02 (d, *J* = 17.2, 4H) 5.30 (s, 1H), 4.15 (d, *J* = 14.0 Hz, 1H), 3.68 (s, 3H), 3.17 (d, *J* = 14.4 Hz, 1H), 2.40 (s, 3H); ^13^C NMR (100 MHz, CDCl_3_) δ 168.5, 167.9, 159.0, 148.0, 145.3, 144.4, 142.1, 135.1, 132.4, 130.2, 130.0, 129.3, 129.0, 128.4, 127.4, 126.8, 117.9, 114.2, 108.7, 102.1, 98.5, 65.9, 55.2, 49.2, 38.2, 21.5; IR: 3352, 2971, 1764, 1668, 1502, 1481, 1332, 1130 cm^−1^; HRMS (ESI): *m*/*z* calcd for C_32_H_27_NNaO_6_ [M + Na]^+^: 544.1736; found 544.1733; Elemental Analysis for C_32_H_27_NO_6_: C, 73.69; H, 5.22; N, 2.69; O, 18.41. Found: C, 73.67; H, 5.23; N, 2.68; O, 18.42.

*N-(7-benzyl-8-(4-methoxyphenyl)-6-oxo-7,8-dihydro-6H-[1,3]dioxolo [4,5-g]chromen-7-yl)-4-(trifluoromethyl)benzamide* (**3d**), 71% yield; ^1^H NMR (400 MHz, CDCl_3_) δ 7.60 (d, *J* = 8.1 Hz, 2H), 7.46 (d, *J* = 8.0 Hz, 2H), 7.23–7.21 (m, 3H), 7.05–7.03 (m, 4H), 6.82 (s, 1H), 6.62–6.70 (m, 4H), 5.97 (d, *J* = 16.8 Hz, 2H), 5.23 (s, 1H), 4.11 (d, *J* = 16 Hz, 1H), 3.70 (s, 3H), 3.20 (d, *J* = 14 Hz, 1H); ^13^C NMR (100 MHz, CDCl_3_) δ 168.4, 166.7, 159.1, 148.2, 145.5, 144.2, 138.3, 134.8, 130.1, 129.9, 128.9, 128.6, 127.6–, 127.2, 125.8, 125.8, 117.5, 114.2, 108.6, 102.1, 98.6, 66.0, 55.3, 49.3, 38.3; ^19^F NMR (376 MHz, CDCl_3_) δ -62.9; IR: 3347, 2981, 1758, 1667, 1502, 1481, 1326, 1130 cm^−1^; HRMS (ESI): *m*/*z* calcd for C_32_H_24_F_3_NNaO_6_ [M + Na]^+^: 598.1453; found 598.1455; Elemental Analysis for C_32_H_24_FNO_6_: C, 66.78; H, 4.20; N, 2.43; O, 16.68. Found: C, 66.76; H, 4.21; N, 2.43; O, 16.67.

*N-(7-benzyl-8-(4-methoxyphenyl)-6-oxo-7,8-dihydro-6H-[1,3]dioxolo [4,5-g]chromen-7-yl)-4-chlorobenzamide* (**3e**), 76% yield; ^1^H NMR (400 MHz, CDCl_3_) δ 7.40–7.37 (m, *J* = 1.8, 4H), 7.20–7.19 (m, *J* = 3.2, 3H), 7.04 (dd, *J* = 8.4, 4H), 6.81 (s, 1H), 6.70–6.86 (m, 4H), 6.02 (dd, *J* = 14.8, 2H), 5.24 (s, 1H), 4.15 (d, *J* = 14.0, 1H), 3.69 (s, 3H), 3.20 (d, *J* = 13.6, 1H); ^13^C NMR (100 MHz, CDCl_3_) δ 168.4, 166.8, 159.1, 148.1, 145.4, 144.3, 138.0, 134.9, 133.4, 130.1, 129.9, 129.0, 128.5, 128.2, 127.5, 117.7, 114.2, 108.7, 102.1, 98.6, 66.0, 55.2, 49.2, 38.3, 25.5; IR: 3355, 2972, 1762, 1666, 1502, 1481, 1332, 1129 cm^−1^; HRMS (ESI): *m*/*z* calcd for C_31_H_24_ClNNaO_6_ [M + Na]^+^: 564.1190; found 564.1196; Elemental Analysis for C_31_H_24_ClNO_6_: C, 68.70; H, 4.46; N, 2.58; O, 17.71. Found: C, 68.69; H, 4.46; N, 2.58; O, 17.72.

*N-(7-benzyl-8-(4-methoxyphenyl)-6-oxo-7,8-dihydro-6H-[1,3]dioxolo [4,5-g]chromen-7-yl)-3-methylbenzamide* (**3f**), 75% yield; ^1^H NMR (400 MHz, CDCl_3_) δ 7.25 (d, *J* = 10.4 Hz, 2H), 7.21– 7.19 (m, 5H), 7.06 (dd, *J* = 14, 6.8 Hz, 4H), 6.80 (s, 1H), 6.70 (dd, *J* = 6.8, 3.2 Hz, 4H), 5.98 (dd, *J* = 16.8, 1.2 Hz, 2H), 5.28 (s, 1H), 4.15 (d, *J* = 15.2 Hz, 1H), 3.68 (s, 3H), 3.17 (d, *J* = 14.0 Hz, 1H), 2.33 (s, 3H); ^13^C NMR (100 MHz, CDCl_3_) δ 168.5, 168.2, 159.0, 148.1, 145.4, 144.3, 138.6, 135.2, 135.1, 132.4, 130.3, 130.0, 129.0, 128.5, 128.5, 127.4, 123.9, 117.9, 114.2, 108.7, 102.1, 98.5, 65.9, 55.2, 49.2, 38.2, 21.4; IR: 3358, 2972, 1774, 1666, 1514, 1476, 1320, 1128 cm^−1^; HRMS (ESI): *m*/*z* calcd for C_32_H_27_NNaO_6_ [M + Na]^+^: 544.1736; found 544.1734; Elemental Analysis for C_32_H_27_NO_6_: C, 73.69; H, 5.22; N, 2.69; O, 18.41. Found: C, 73.68; H, 5.23; N, 2.68; O, 18.42.

*N-(7-benzyl-8-(4-methoxyphenyl)-6-oxo-7,8-dihydro-6H-[1,3]dioxolo [4,5-g]chromen-7-yl)-2-chlorobenzamide* (**3g**), 68% yield; ^1^H NMR (400 MHz, CDCl_3_) δ 7.45–7.40 (m, 2H), 7.20–7.17 (m, 3H), 7.05–7.01 (m, 4H), 6.82 (d, *J* = 8.8 Hz, 2H), 6.80 (s, 1H), 6.69–6.68 (m, 3H), 6.64 (s, 1H), 5.99 (dd, *J* = 16.0, 1.6 Hz, 2H), 5.27 (s, 1H), 4.13 (d, *J* = 14.0 Hz, 1H), 3.80 (s, 3H), 3.67 (s, 3H), 3.13 (d, *J* = 14.0 Hz, 1H); ^13^C NMR (100 MHz, CDCl_3_) δ 168.3, 166.3, 159.2, 148.1, 145.4, 144.1, 134.8, 134.6, 131.6, 130.7, 130.4, 130.2, 129.6, 129.2, 128.5, 127.5, 126.9, 117.8, 114.4, 108.6, 102.1, 98.6, 66.1, 55.4, 49.6, 38.6; IR: 3359, 2971, 1764, 1666, 1502, 1482, 1324, 1130 cm^−1^; HRMS (ESI): *m*/*z* calcd for C_31_H_24_ClNNaO_6_ [M + Na]^+^: 564.1190; found 564.1187; Elemental Analysis for C_31_H_24_ClNO_6_: C, 68.70; H, 4.46; N, 2.58; O, 17.71. Found: C, 68.69; H, 4.46; N, 2.58; O, 17.72.

*N-(7-benzyl-8-(4-methoxyphenyl)-6-oxo-7,8-dihydro-6H-[1,3]dioxolo [4,5-g]chromen-7-yl)-3,5-dimethylbenzamide* (**3h**), 69% yield; ^1^H NMR (400 MHz, CDCl_3_) δ 7.30–7.22 (m, 3H), 7.06–6.86 (m, 7H), 6.81 (s, 1H), 6.69 (dd, *J* = 46.8, 2.8 Hz, 4H), 5.99 (d, *J* = 15.6 Hz, 2H), 5.28 (s, 1H), 4.12 (d, *J* = 14.0 Hz, 1H), 3.70 (s, 3H), 3.17 (d, *J* = 13.6 Hz, 1H), 2.28 (s, 6H); ^13^C NMR (100 MHz, CDCl_3_) δ 168.5, 168.3, 159.0, 148.0, 145.4, 144.4, 138.4, 135.2, 135.1, 133.3, 130.3, 130.0, 129.0, 128.4, 127.4, 124.5, 117.9, 114.2, 108.7, 102.1, 98.5, 65.9, 55.2, 49.2, 38.2, 21.3; IR: 3357, 2973, 1764, 1666, 1502, 1481, 1345, 1142 cm^−1^; HRMS (ESI): *m*/*z* calcd for C_33_H_29_NNaO_6_ [M + Na]^+^: 558.1893; found 558.1895.

*N-(7-(4-fluorobenzyl)-8-(4-methoxyphenyl)-6-oxo-7,8-dihydro-6H-[1,3]dioxolo [4,5-g]chromen-7-yl)benzamide* (**3i**), 72% yield; ^1^H NMR (400 MHz, CDCl_3_) 7.40 (dd, *J* = 34.6, 6.5 Hz, 5H), 7.16–6.46 (m, 11H), 5.99 (d, *J* = 15.1 Hz, 2H), 5.26 (s, 1H), 4.12 (d, *J* = 14.0 Hz, 1H), 3.67 (s, 3H), 3.14 (d, *J* = 14.0 Hz, 1H); ^13^C NMR (100 MHz, CDCl_3_) δ 168.4, 167.9, 159.0, 148.0, 145.4, 144.2, 134.9, 131.7, 131.4, 131.3, 130.7, 130.0, 128.9, 128.7, 126.7, 117.6, 115.4, 115.2, 114.2, 108.6, 102.0, 98.4, 65.8, 49.1, 37.4, 25.4; ^19^F NMR (376 MHz, CDCl_3_) δ -114.9; IR: 3360, 2971, 1758, 1672, 1500, 1480, 1322, 1123 cm^−1^; HRMS (ESI): *m*/*z* calcd for C_31_H_24_FNNaO_6_ [M + Na]^+^: 548.1485; found 548.1489.

*N-(7-(4-chlorobenzyl)-8-(4-methoxyphenyl)-6-oxo-7,8-dihydro-6H-[1,3]dioxolo [4,5-g]chromen-7-yl)benzamide* (**3j**), 71% yield; ^1^H NMR (400 MHz, CDCl_3_) δ 7.43 (d, *J* = 6.7 Hz, 3H), 7.36 (d, *J* = 6.7 Hz, 2H), 7.16 (d, *J* = 7.4 Hz, 2H), 7.05 (dd, *J* = 19.2, 8.8 Hz, 4H), 6.80 (s, 1H) 6.68 (m, 4H), 5.98 (d, *J* = 15.6 Hz, 2H), 5.25 (s, 1H), 4.14 (d, *J* = 14.4 Hz, 1H), 3.68 (s, 3H), 3.15 (d, *J* = 13.9 Hz, 1H); ^13^C NMR (100 MHz, CDCl_3_) δ 168.3, 168.0, 159.1, 148.1, 145.5, 144.3, 134.9, 133.5, 133.4, 131.8, 131.2, 130.0, 129.0, 128.8, 128.7, 126.7, 117.6, 114.2, 108.7, 102.1, 98.5, 65.8, 55.2, 49.1, 37.6; IR: 3348, 2976, 1764, 1666, 1502, 1480, 1324, 1102 cm^−1^; HRMS (ESI): *m*/*z* calcd for C_31_H_24_ClNNaO_6_ [M + Na]^+^: 564.1190; found 564.1187; Elemental Analysis for C_31_H_24_ClNO_6_: C, 68.70; H, 4.46; N, 2.58; O, 17.71. Found: C, 68.69; H, 4.46; N, 2.58; O, 17.72.

*N-(8-(4-methoxyphenyl)-7-(2-(methylthio)ethyl)-6-oxo-7,8-dihydro-6H-[1,3]dioxolo [4,5-g]chromen-7-yl)benzamide* (**3k**), 65% yield; ^1^H NMR (400 MHz, CDCl_3_) δ 7.55–7.53 (m, 2H), 7.51–7.47 (m, 1H), 7.41–6.37 (m, 2H), 7.00–6.97 (m, 2H), 6.92 (s, 1H), 6.71–6.68 (m, 3H),6.63 (s, 1H), 5.99 (d, *J* = 15.4 Hz, 2H), 5.09 (s, 1H), 3.69 (s, 3H), 3.25–3.17 (m, 1H), 2.51 (td, *J* = 12.7, 5.0 Hz, 1H), 2.35 (td, *J* = 12.4, 4.9 Hz, 1H), 2.18–2.09 (m, 1H), 2.01 (s, 3H); ^13^C NMR (100 MHz, CDCl_3_) δ 169.2, 167.4, 159.1, 148.0, 145.4, 144.3, 134.6, 131.9, 130.0, 128.9, 128.8, 126.9, 117.4, 114.2, 108.6, 102.1, 98.5, 64.2, 55.3, 49.1, 32.6, 29.0, 15.7; IR: 3362, 2972, 1724, 1663, 1502, 1481, 1324, 1104 cm^−1^; HRMS (ESI): *m*/*z* calcd for C_27_H_25_NNaO_6_S [M + Na]^+^: 514.1300; found 514.1305.

*N-(7-isobutyl-8-(4-methoxyphenyl)-6-oxo-7,8-dihydro-6H-[1,3]dioxolo [4,5-g]chromen-7-yl)benzamide* (**3l**), 66% yield; ^1^H NMR (400 MHz, CDCl_3_) δ 7.48–7.56 (m, 3H), 7.40 (t, *J* = 7.4 Hz, 2H), 6.98–7.01 (m, 2H), 6.93 (s, 1H), 6.68–6.71 (m, 3H), 6.64 (s, 1H), 6.00 (dd, *J* = 14.7, 1.4 Hz, 2H), 5.09 (s, 1H), 3.70 (s, 3H), 3.49 (d, *J* = 4.8 Hz, 2H), 2.20–2.11 (m, 1H), 0.90 (dd, *J* = 7.2, 20.8 Hz, 6H); ^13^C NMR (100 MHz, CDCl_3_) δ 169.5, 167.6, 159.3, 148.3, 145.6, 144.5, 134.9, 132.1, 130.2, 129.2, 129.0, 127.1, 117.6, 114.5, 108.8, 102.3, 98.8, 64.4, 55.5, 41.04, 21.82, 20.18; IR: 3349, 2970, 1766, 1660, 1500, 1485, 1324, 1102 cm^−1^; HRMS (ESI): *m*/*z* calcd for C_28_H_27_NNaO_6_ [M + Na]^+^: 496.1736; found 496.1740.

*N-(8-(4-methoxyphenyl)-6-oxo-7-phenyl-7,8-dihydro-6H-[1,3]dioxolo [4,5-g]chromen-7-yl)benzamide* (**3m**), 67% yield; ^1^H NMR (400 MHz, CDCl_3_) δ 7.38–7.52 (m, 3H), 7.29–7.34 (m, 2H), 7.13–7.22 (m, 7H), 7.04–7.09 (m, 2H), 6.82 (s, 1H), 6.70 (d, *J* = 6.4 Hz, 2H), 6.01 (dd, *J* = 16.7, 1.1 Hz, 2H), 5.32 (s, 1H), 3.69 (s, 3H); ^13^C NMR (100 MHz, CDCl_3_) δ 168.7, 168.5, 148.5, 145.7, 144.8, 138.6, 135.6, 135.3, 132.0, 130.4, 129.3, 129.0, 128.9, 128.2, 127.8, 127.1, 117.9, 109.1, 102.5, 99.0, 60.1, 50.5, 38.7; IR: 3344, 2975, 1760, 1660, 1500, 1485, 1324, 1092 cm^−1^; HRMS (ESI): *m*/*z* calcd for C_30_H_23_NNaO_6_ [M + Na]^+^: 516.1423; found 516.1427.

*N-(7-benzyl-6-oxo-8-phenyl-7,8-dihydro-6H-[1,3]dioxolo [4,5-g]chromen-7-yl)benzamide* (**3n**), 64% yield; ^1^H NMR (400 MHz, CDCl_3_) δ 7.47–7.43 (m, 3H), 7.36–7.32 (m, 2H), 7.21–7.19 (m, 3H), 7.07–7.04 (m, 4H), 6.81 (s, 1H), 6.71–6.68 (m, 4H), 6.01 (d, *J* = 15.4 Hz, 2H), 4.13 (d, *J* = 14.0 Hz, 1H), 3.16 (d, *J* = 14 Hz, 1H); ^13^C NMR (100 MHz, CDCl_3_) δ 168.3, 168.1, 148.2, 145.4, 144.4, 138.3, 135.2, 134.9, 131.6, 130.0, 128.9, 128.6, 128.5, 127.9, 127.5, 126.7, 117.5, 108.7, 102.1, 98.6, 65.7, 50.1, 38.3; IR: 3354, 2974, 1762, 1626, 1508, 1488, 1324, 1120 cm^−1^; HRMS (ESI): *m*/*z* calcd for C_30_H_23_NNaO_5_ [M + Na]^+^: 500.1474; found 500.1471.

*N-(7-benzyl-8-((E)-4-methoxystyryl)-6-oxo-7,8-dihydro-6H-[1,3]dioxolo [4,5-g]chromen-7-yl)benzamide* (**3o**), 72% yield; ^1^H NMR (400 MHz, CDCl_3_) δ 7.65–7.61 (m, 2H), 7.51–7.46 (m, 1H), 7.41–7.37 (m, 2H), 7.22–7.20 (m, 3H), 7.15–7.11 (m, 2H), 7.04–7.00 (m, 2H), 6.77 (d, *J* = 5.6 Hz, 2H), 6.74–6.72 (m, 2H), 6.35 (d, *J* = 16 Hz, 1H), 6.04 (d, *J* = 4.4 Hz, 2H), 5.89 (dd, *J* = 15.6, 8.0 Hz, 1H), 3.91 (d, *J* = 14 Hz, 1H), 3.74 (s, 3H), 3.06 (d, *J* = 14.4 Hz, 1H); ^13^C NMR (100 MHz, CDCl_3_) δ 168.5, 167.4, 159.3, 148.0, 145.2, 144.1, 134.8, 132.8, 131.7, 129.9, 129.1, 128.7, 128.4, 127.7, 127.4, 126.8, 123.0, 116.6, 113.8, 108.6, 102.0, 98.6, 64.9, 53.5, 47.1, 37.7; IR: 3386, 2978, 1784, 1672, 1500, 1481, 1332, 1135 cm^−1^; HRMS (ESI): *m*/*z* calcd for C_33_H_27_NNaO_6_ [M + Na]^+^: 556.1736; found 556.1731.

*The gram-scale synthesis of dihydrocoumarin* **3a***:* To a stirred solution of 2-benzylphenol (**1**, 0.517 g, 2.0 mmol), 4-benzyl-2-phenyloxazol-5(4*H*)-one (**2**, 0.503 g, 2.0 mmol), and Ag_2_O (0.556 g, 2.4 mmol) in chloroform (30 mL), diphenyl phosphate (50.0 mg, 0.2 mmol) was added at room temperature under an N_2_ atmosphere. The reaction mixture was stirred for 62 h at room temperature. After completion of the reaction (monitored by TLC), the solvent was removed under reduced pressure. After extraction with ethyl acetate (90 mL) three times and drying with anhydrous sodium sulfate, the resulting solution was concentrated by an evaporator and purified by silica gel column chromatography (EtOAc/hexane = 1:5) to afford *N*-(7-benzyl-8-(4-methoxyphenyl)-6-oxo-7,8-dihydro-6*H*-[1,3]dioxolo [4,5-g]chromen-7-yl)benzamide **3a** (0.721 g, 78%).

*The typical procedure for the asymmetric synthesis of 3,4-dihydrocoumarins* **3a***:* To a stirred solution of 2-benzylphenol (**1**, 25.8 mg, 0.1 mmol), 4-benzyl-2-phenyloxazol-5(4H)-one (**2**, 25.1 mg, 0.1 mmol), and Ag_2_O (27.8 mg, 0.12 mmol) in chloroform (2 mL), (11bR)-2,6-di(anthracen-9-yl)-4-hydroxydinaphtho [2,1-d:1’,2’-f][1–3]dioxaphosphepine 4-oxide (**4**, 7.0 mg, 0.01 mmol) was added at room temperature under an N_2_ atmosphere. The reaction mixture was stirred for 42 h at room temperature. After completion of the reaction (monitored by TLC), the solvent was removed under reduced pressure. The crude product was purified by flash chromatography (EtOAc/hexane = 1:5) to afford *N*-((*7S,8S*)-7-benzyl-8-(4-methoxyphenyl)-6-oxo-7,8-dihydro-6*H*-[1,3]dioxolo [4,5-g]chromen-7-yl)benzamide **3a** (31.9 mg).

63% yield; [α${]}_{D}^{25}$ = +8.01 (c = 0.20, CH_2_Cl_2_); ^1^H NMR (400 MHz, CDCl_3_) δ 7.45–7.41 (m, 2H), 7.20–7.16 (m, 3H), 7.05–7.02 (m, 4H), 6.82 (d, *J* = 8.8 Hz, 2H), 6.80 (s, 1H), 6.69–6.68 (m, 3H), 6.64 (s, 1H), 5.99 (dd, *J* = 16.0, 1.6 Hz, 2H), 5.27 (s, 1H), 4.13 (d, *J* = 14.0 Hz, 1H), 3.67 (s, 3H), 3.13 (d, *J* = 14.1 Hz, 1H); ^13^C NMR (100 MHz, CDCl_3_) δ 168.5, 168.0, 159.0, 148.1, 144.4, 135.2, 135.0, 131.7, 130.2, 129.9, 129.0, 128.7, 128.5, 127.4, 126.8, 117.8, 114.2, 108.7, 102.1, 98.5, 77.4, 77.1, 76.8, 66.0, 55.2, 49.2, 38.2; IR: 3356, 2970, 1764, 1666, 1502, 1481, 1324, 1128 cm^−1^; Elemental Analysis for C_31_H_25_NO_6_: C, 73.36; H, 4.96; N, 2.76; O, 18.91. Found: C, 73.34; H, 4.98; N, 2.75; O, 18.93; HRMS (ESI): *m*/*z* calcd for C_31_H_25_NNaO_6_ [M + Na]^+^: 530.1580; found 530.1585; The EE value was 93%, tR (major) = 36.40 min, tR (minor) = 30.45 min (Daicel chiralcel IA, λ = 254 nm, n-hexane/i-PrOH 97/3, 1.0 mL/min).

## 4. Conclusions

In summary, we have developed an efficient synthesis of 3,4-dihydrocoumarin derivatives via C–H oxidation between 2-alkyl substituted phenol derivatives and 5-(4*H*)oxazolones and cyclization cascade under acid-catalyzed conditions. This approach provides a one-pot strategy to synthesize multisubstituted 3,4-dihydrocoumarins in moderate to high yields (64–81%) and excellent diastereoselectivity (>20:1).

## Figures and Tables

**Table 1:**
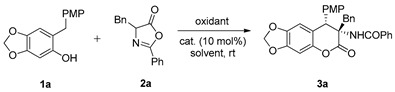
Optimization of the reaction conditions ^[a,c]^.

Entry	Oxidant	Catalyst	Solvent	Yield ^[b]^ (%)
1	Ag_2_CO_3_	*p*-TsOH	CHCl_3_	35
2	Ag_2_O	*p*-TsOH	CHCl_3_	63
3	AgOAc	*p*-TsOH	CHCl_3_	25
4	AgOTf	*p*-TsOH	CHCl_3_	33
5	AgBF_4_	*p*-TsOH	CHCl_3_	28
6	AgNO_3_	*p*-TsOH	CHCl_3_	18
7	MnO_2_	*p*-TsOH	CHCl_3_	41
8	Mn(OAc)_3_	*p*-TsOH	CHCl_3_	29
9	Mn(acac)_3_	*p*-TsOH	CHCl_3_	61
10	K_2_S_2_O_8_	*p*-TsOH	CHCl_3_	trace
11	Oxone	*p*-TsOH	CHCl_3_	trace
12	TBHP	*p*-TsOH	CHCl_3_	trace
13	DDQ	*p*-TsOH	CHCl_3_	trace
14	PhI(OAc)_2_	*p*-TsOH	CHCl_3_	trace
15	PIFA	*p*-TsOH	CHCl_3_	trace
16	Ag_2_O	MsOH	CHCl_3_	61
17	Ag_2_O	DNBS	CHCl_3_	57
18	Ag_2_O	AcOH	CHCl_3_	15
19	Ag_2_O	TFA	CHCl_3_	45
20	Ag_2_O	DPP	CHCl_3_	81
21	Ag_2_O	DPP	CH_2_Cl_2_	72
22	Ag_2_O	DPP	DCE	70
23	Ag_2_O	DPP	EtOAc	46
24	Ag_2_O	DPP	EtOEt	38
25	Ag_2_O	DPP	THF	58
26	Ag_2_O	DPP	dioxane	47
27	Ag_2_O	DPP	benzene	38
28	Ag_2_O	DPP	toluene	45
29	Ag_2_O	DPP	*p*-xylene	42

^[a]^ Reaction conditions: benzyl phenol **1a** (0.2 mmol), oxazolone **2a** (0.2 mmol), oxidant (0.24 mmol), and catalyst (0.02 mmol) in solvent (3 mL) at room temperature for 24 h under N_2_ atmosphere. ^[b]^ Isolated yield. ^[c]^ The relative configuration of the indicated diastereomer was determined by ^1^H-NMR analysis. PMP = *para*-methoxyphenyl, TBHP = *tert-*butyl hydroperoxide, DDQ = 2,3-dichloro-5,6-dicyano-1,4-benzoquinone, Hacac = acetylacetone, PIFA = [bis(trifluoroacetoxy)iodo]benzene.

**Table 2:** Substrate scope ^[a,b,c]^.

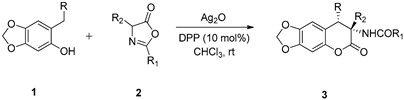
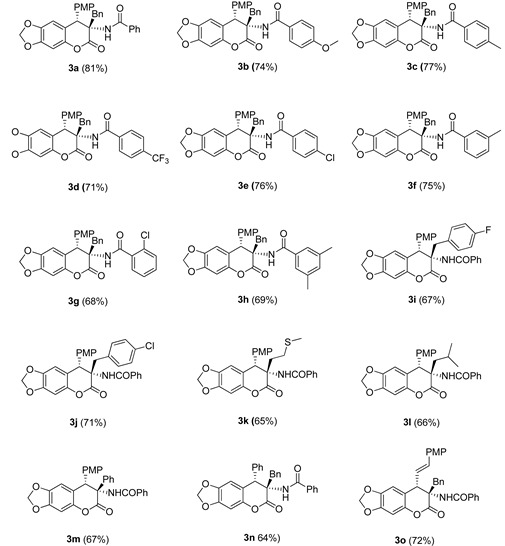

^[a]^ Reaction conditions: benzyl phenol **1** (0.2 mmol), oxazolone **2** (0.2 mmol), Ag_2_O (0.24 mmol), and diphenyl phosphate (0.01 mmol) in chloroform (3 mL) at room temperature for 24–36 h under N_2_ atmosphere. ^[b]^ Isolated yield. ^[c]^ The relative configuration of the indicated diastereomer was determined by ^1^H-NMR analysis.

## Data Availability

Data is contained within the article or Supplementary Materials.

## Supplementary Materials

The following supporting information can be downloaded at: https://www.mdpi.com/article/10.3390/molecules28196853/s1. The experimental procedures and ^1^H NMR, and ^13^C NMR spectra of products can be found in the supporting information. References are cited from [36,37,38,39,40,41,42].
