# Supplementary material for: One-Pot Synthesis of 3,4-Dihydrocoumarins via C-H Oxidation/Conjugate Addition/Cyclization Cascade Reaction
Source: Molecules. 2023 Sep 28;28(19):6853. doi: 10.3390/molecules28196853 (PMC10574682; doi:10.3390/molecules28196853)
Supplement: Supplementary file 1 [file molecules-28-06853-s001.zip › molecules-2631592-supplementary.pdf]

Supporting Information

One-pot Synthesis of 3,4-Dihydrocoumarins via C-H Oxidation/Conjugate Addition/Cyclization Cascade Reaction

Dae Young Kim

Department of Chemistry, Soonchunhyang University, Asan, Chungnam 31538, Korea

Contents

1. General information --- S2

2. Preparation of starting materials -- S2

3. Reference --- S2

4. NMR spectra -- S3

1. General information

All chemicals were purchased from commercial suppliers and used without further purification unless otherwise specified. Solvents for extractions and chromatography were of technical grade and were distilled prior to use. Extracts were dried over technical-grade anhydrous Na_2SO_4. Anhydrous solvents were deoxygenated by sparging with N_2 and dried by passing through activated alumina columns of a Pure Solv solvent purification system (Innovative Technology). Reactions were monitored by analytical thin-layer chromatography (TLC) using silica gel 60 F_{254} pre-coated glass plates (0.25 mm thickness) and visualized using UV light (254 nm and 365 nm), I_2, *p*-anisaldehyde, ninhydrin, and phosphomolybdic acid solution as an indicator. Flash chromatography was carried out on E. Merck silica gel (230–400 mesh). ^{1}H NMR,^{13}C NMR, and ^{19}F NMR spectra were recorded at 400 MHz, 100 MHz, 376 MHz respectively, on a Jeol ECS 400 MHz NMR spectrometer. Chemicals shift values (□) are reported in parts per million and referenced in relation to the following standards: Me_4Si as the internal references for ^{1}H- and ^{13}C-NMR signals in chloroform and $PhCF_3$ as the external references for ^{19}F-NMR signal. The peak information is described as: s = singlet, d = doublet, t = triplet, q = quartet, m= multiplet. Mass spectra (MS-EI, 70 eV) were conducted on GC-MS Shimadzu QP2010. High resolution mass spectra were measured on Jeol HX110/110A using electrospray ionization technique. The enantiomeric excesses (ee's) were determined by HPLC. HPLC analysis was performed on Shimadzu prominence 20 measured at 254 nm using the indicated chiral column. Optical rotations were measured on a JASCO-DIP-1000 digital polarimeter with a sodium lamp. Infrared spectra were recorded on a ThermoFisher Scientific Nicolet iS5 FT-IR spectrometer spectrometer. The elemental analysis was carried out on a Perkin-Elmer 2400 Series II Elemental Analyzer.

2. Preparation of starting materials

2-(4-Methoxybenzyl)phenol derivatives (**1a**)[36], *o*-benzylphenol derivatives (**1l**)[37-39], *o*-Cinnamylphenol derivatives (**4**)[40] were prepared in accordance with literature methods. Oxazolone derivatives (**2a-2j**)[41] were prepared in accordance with literature methods. Bifunctional organocatalysts **I-III** were synthesized in accordance with previously reported papers.[42]

3. References

36. H. Lam, Z. Qureshi, M.Wegmann, M. Lautens, *Angew. Chem.,Int. Ed.* **2018**, *57*, 16185.
37. Y. F. Wong, Z. Wang, W. X. Hong, J.Sun, J. *Tetrahedron* **2016**, *72*, 2748.
38. B. Wu, X. Gao, Z. Yan, M. W. Chen, Y. Z. Zhou, *Org. Lett.* **2015**, *17*, 6134.
39. W. Xiao, Y. Mo, J. Guo, Z. Su, S. Dong, X. Feng, *Chem. Sci.* **2021**, *12*, 2940.
40. A. Adili, Z. L. Tao, D. F. Chen, Z. Y. Han, *Org. Biomol. Chem.* **2015**, *13*, 2247.
41. A. R. Alba, R. Rios, *Chem.-Asian J.* **2011**, *6*, 720.
42. S. M. Kim, J. H. Lee, D. Y. Kim, *Synlett* **2008**, 2659.

4. **NMR spectra**

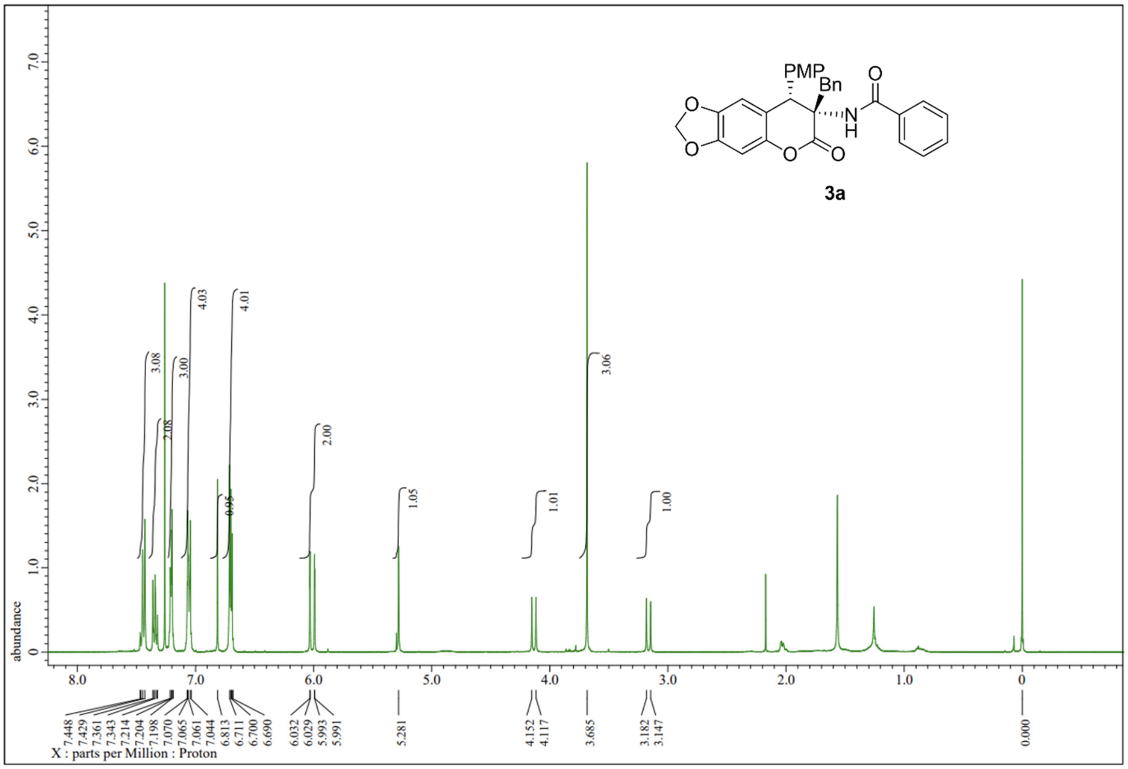

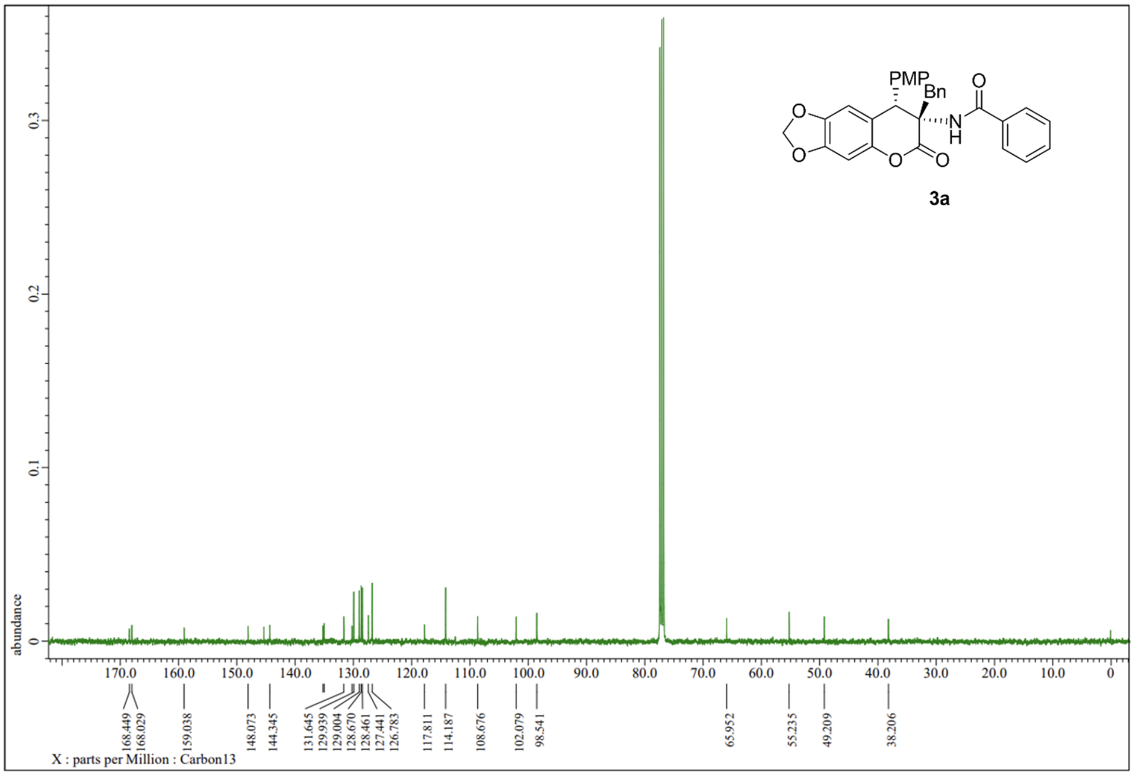

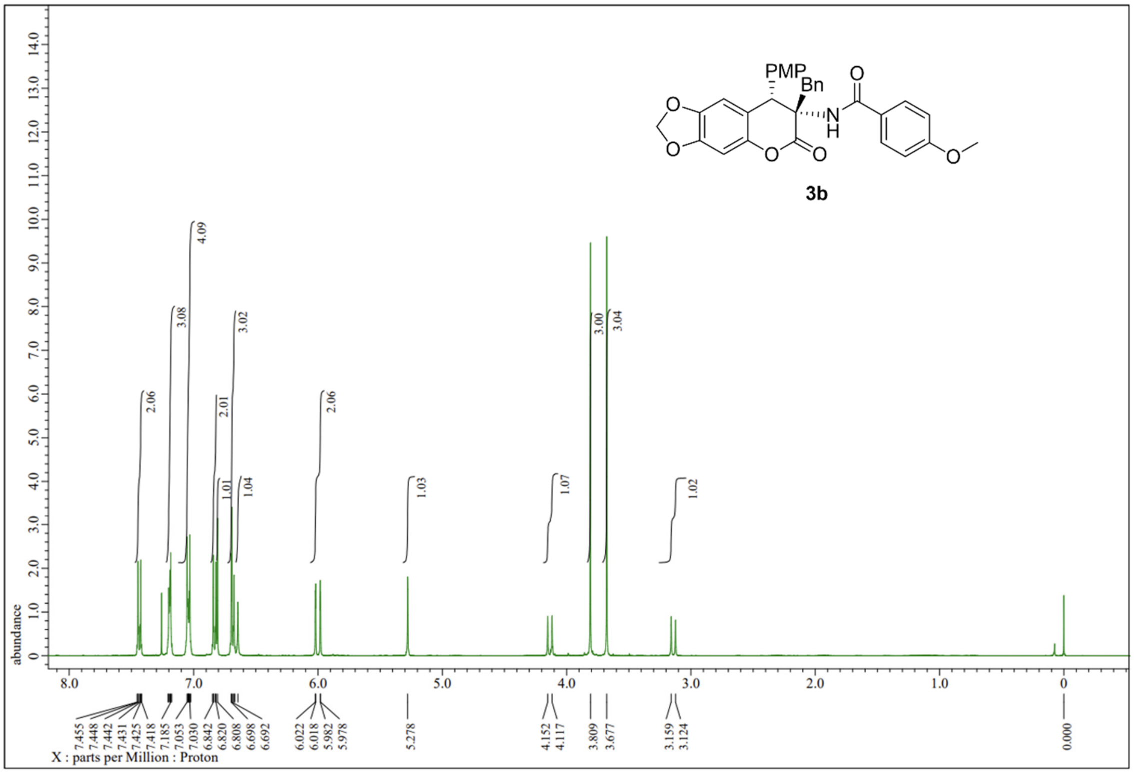

PMP
Bn
3b
abundance
X : parts per Million : Proton
7.455
7.448
7.442
7.431
7.425
7.418
7.185
7.053
7.030
6.842
6.820
6.808
6.698
6.692
6.022
6.018
5.982
5.978
5.278
4.152
4.117
3.809
3.677
3.159
3.124
0.000
2.06
3.08
4.09
2.01
1.01
3.02
1.04
2.06
1.03
1.07
3.00
3.04
1.02

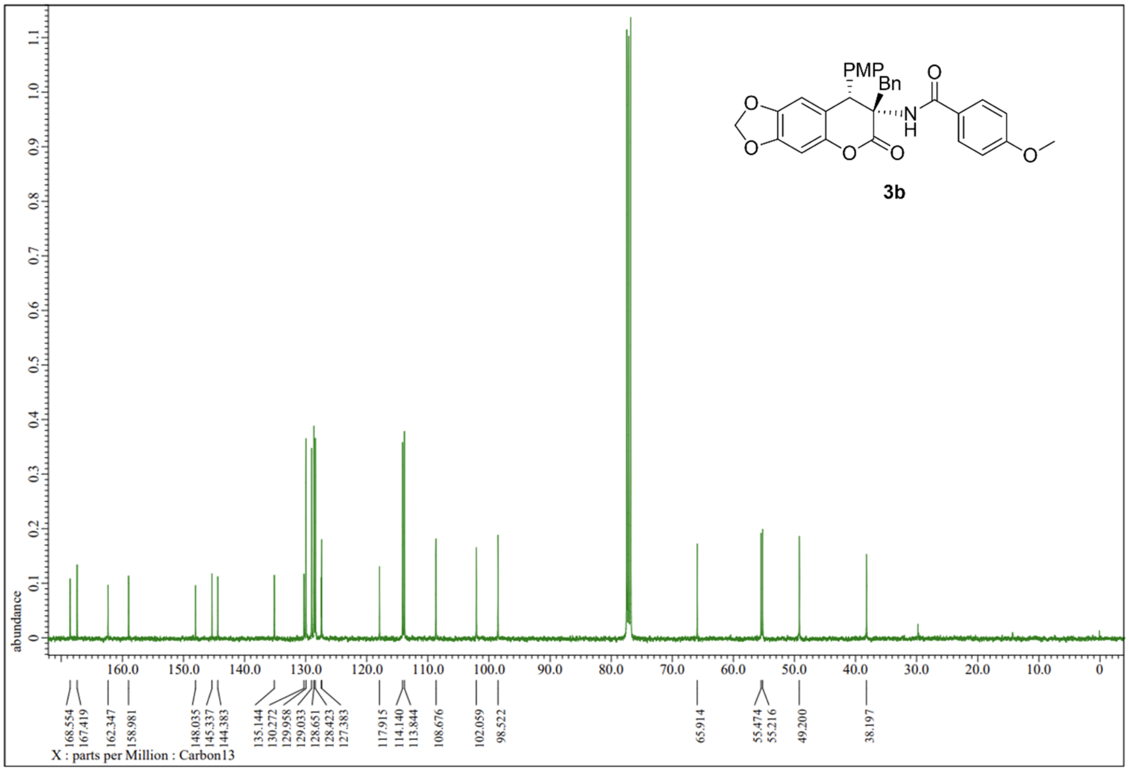

PMP
Bn
3b
abundance
X : parts per Million : Carbon13
168.554
167.419
162.347
158.981
148.035
145.337
144.383
135.144
130.272
129.958
129.033
128.651
128.423
127.383
117.915
114.140
113.844
108.676
102.059
98.522
65.914
55.474
55.216
49.200
38.197

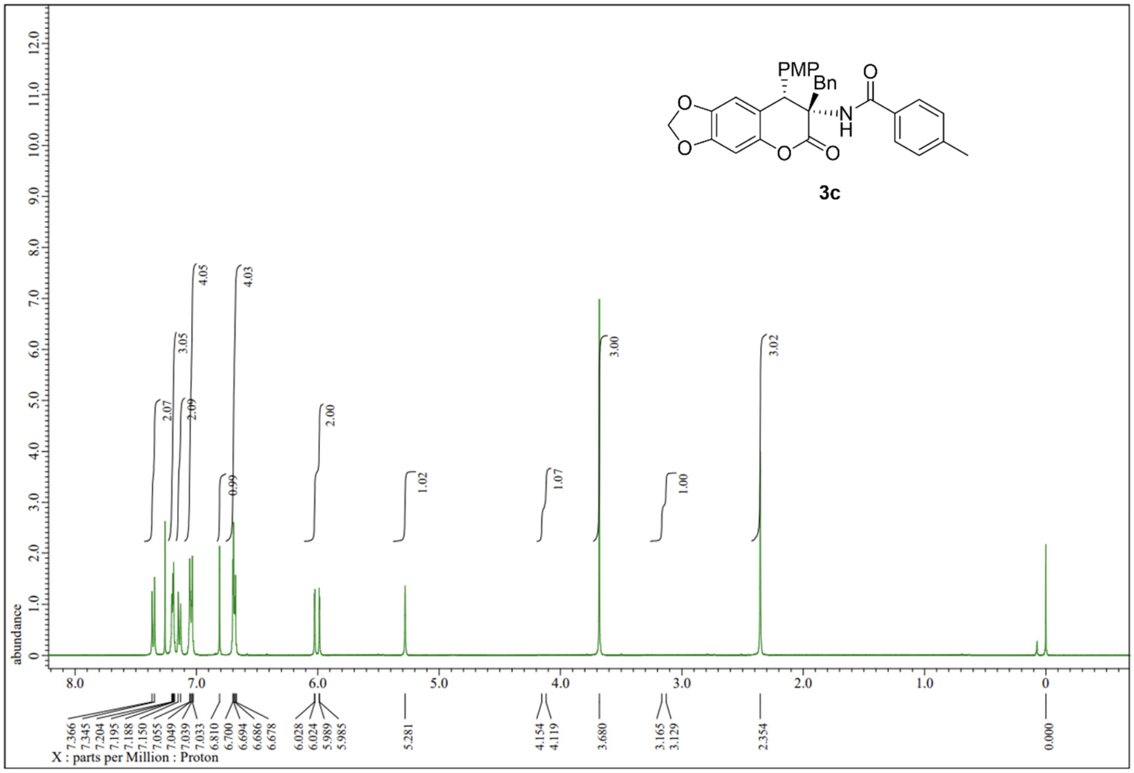

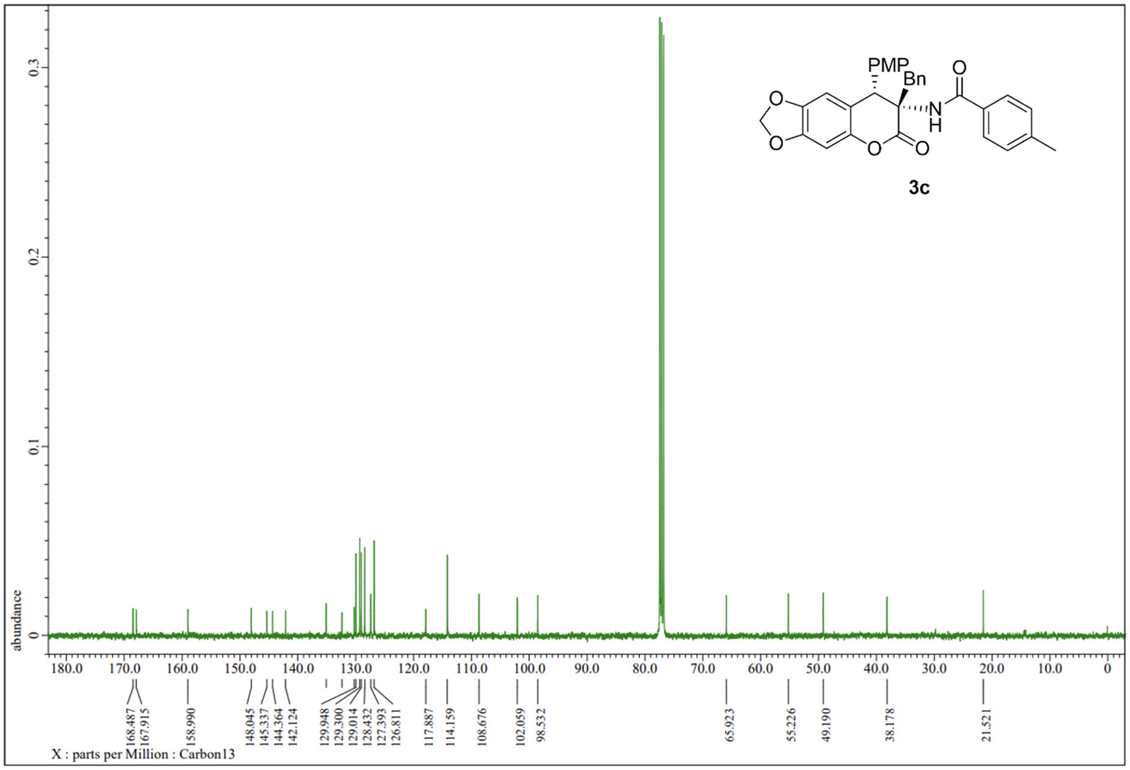

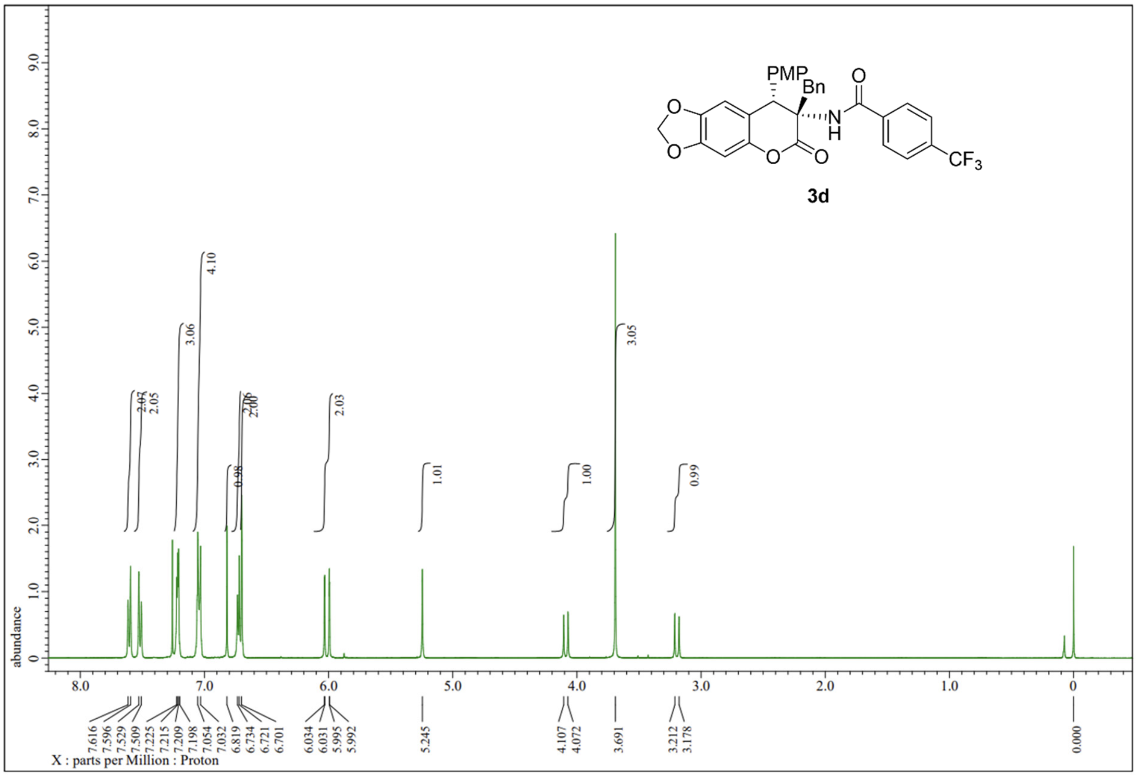
PMP
Bn
O
N
H
O
O
O
O
CF3
3d
abundance
X : parts per Million : Proton
7.616
7.596
7.529
7.509
7.225
7.215
7.209
7.198
7.054
7.032
6.819
6.734
6.721
6.701
6.034
6.031
5.995
5.992
5.245
4.107
4.072
3.691
3.212
3.178
0.000
2.07
2.05
3.06
4.10
0.98
2.06
2.00
2.03
1.01
1.00
3.05
0.99

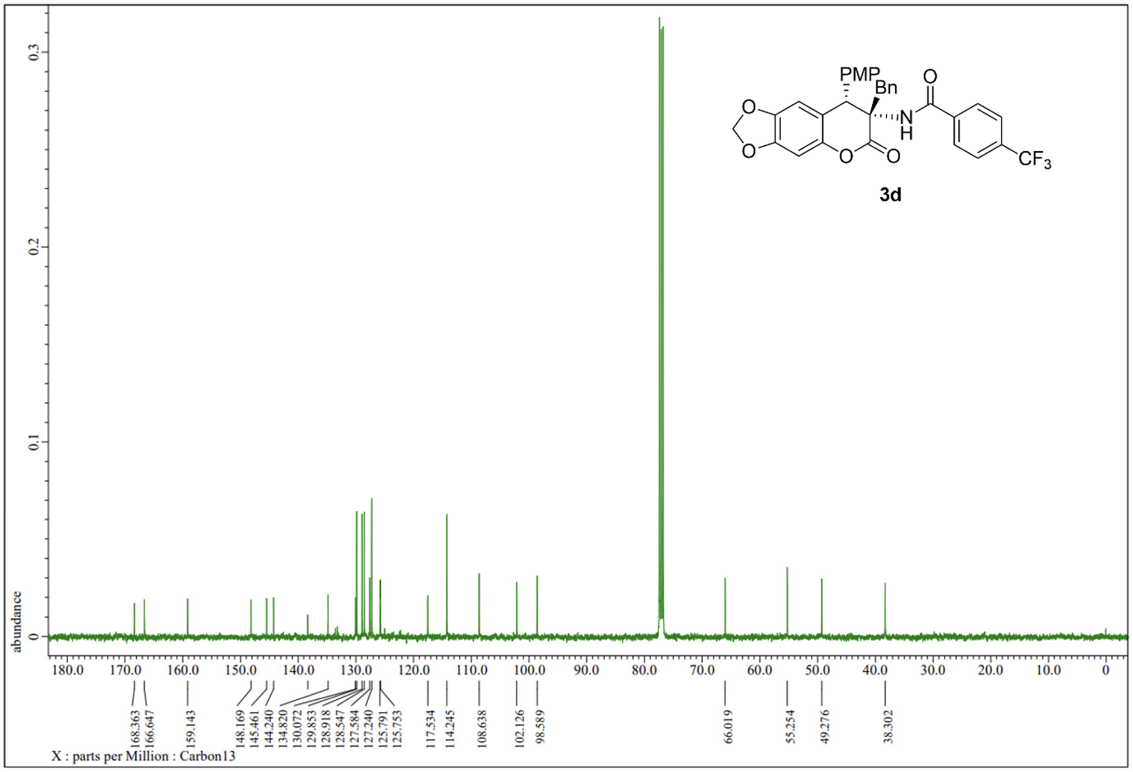
PMP
Bn
O
N
H
O
O
O
O
CF3
3d
abundance
X : parts per Million : Carbon13
168.363
166.647
159.143
148.169
145.461
144.240
134.820
130.072
129.853
128.918
128.547
127.584
127.240
125.791
125.753
117.534
114.245
108.638
102.126
98.589
66.019
55.254
49.276
38.302

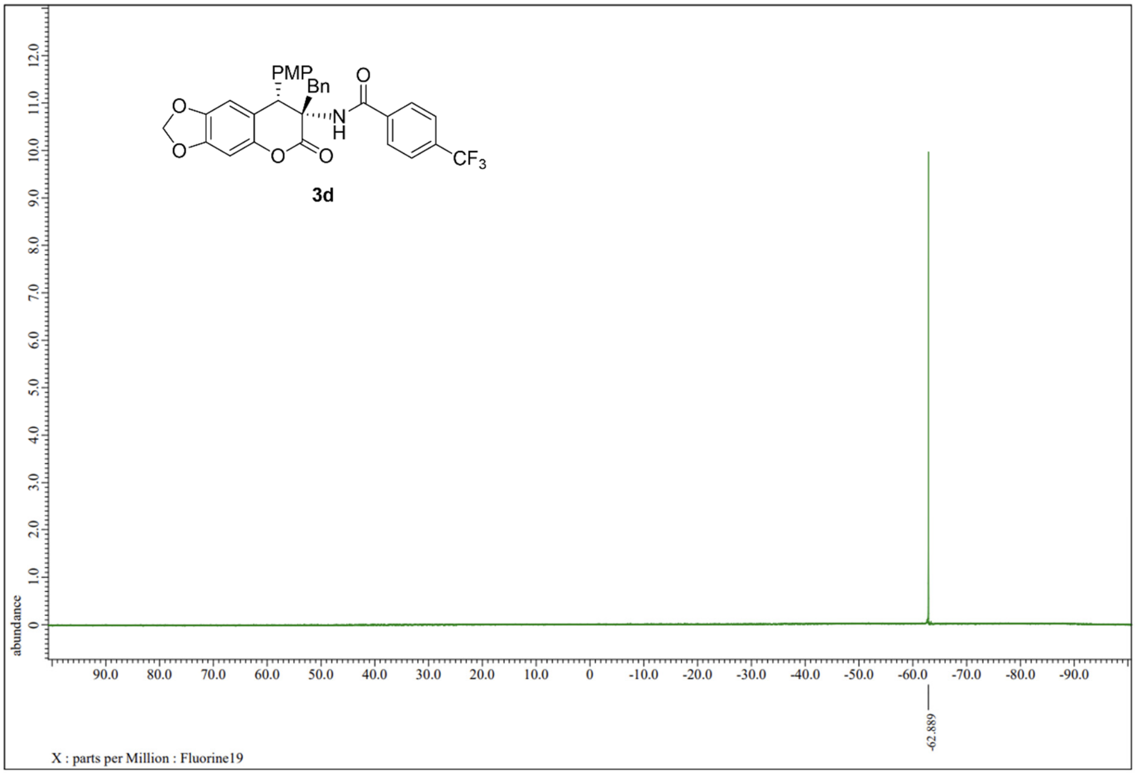

PMP
Bn
3d
CF3
-62.889
X : parts per Million : Fluorine19
abundance

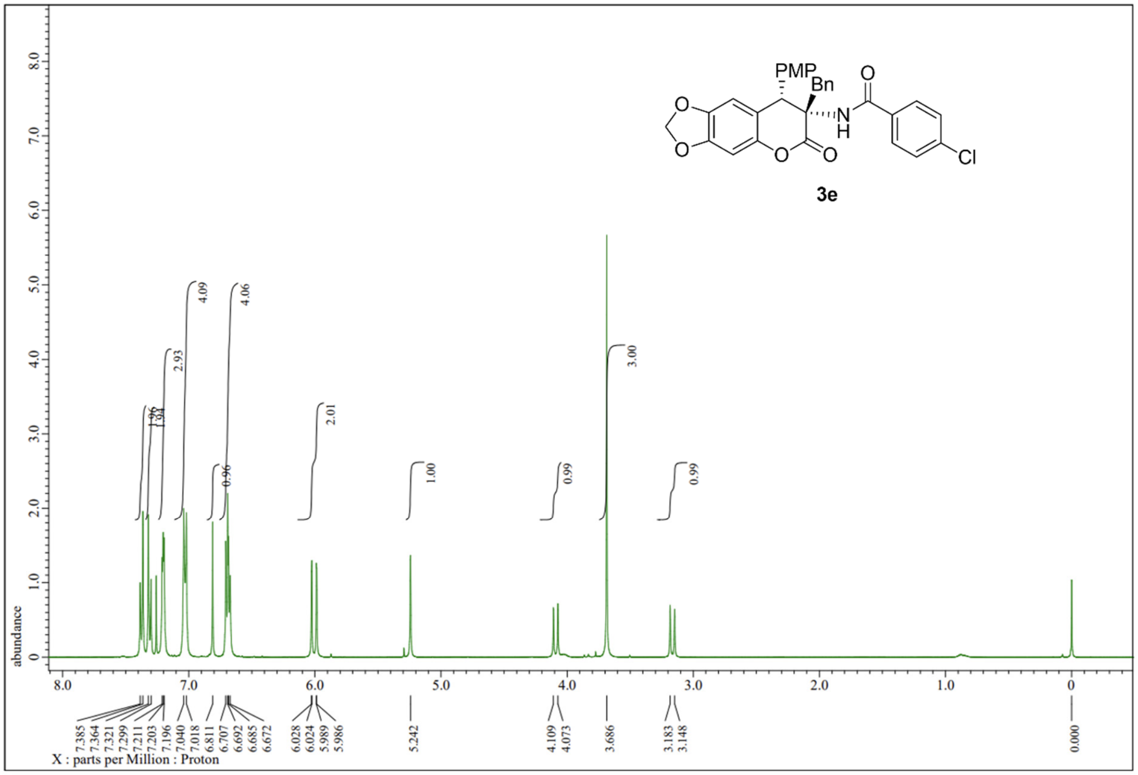

PMP
Bn
3e
Cl
4.09
4.06
2.93
3.00
2.01
1.00
0.99
0.99
7.385
7.364
7.321
7.299
7.211
7.203
7.196
7.040
7.018
6.811
6.707
6.692
6.685
6.672
6.028
6.024
5.989
5.986
5.242
4.109
4.073
3.686
3.183
3.148
0.000
X : parts per Million : Proton
abundance

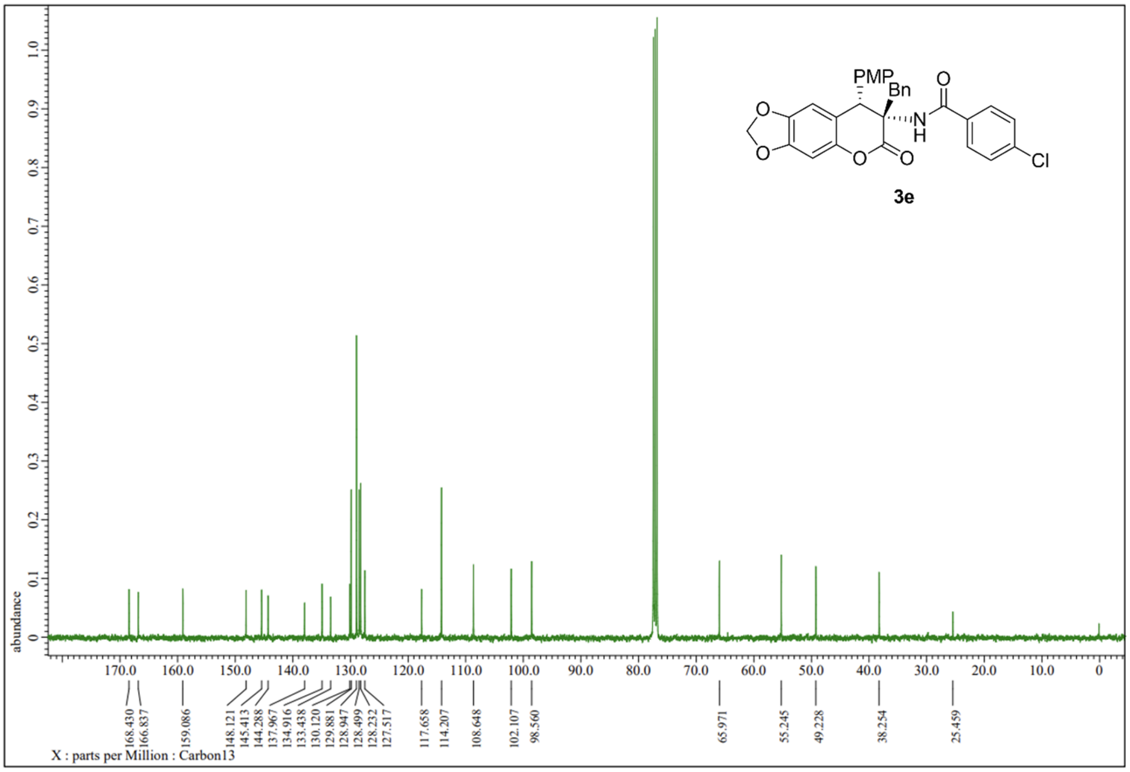
PMP
Bn
O
N
H
Cl
3e
abundance
X : parts per Million : Carbon13
168.430
166.837
159.086
148.121
145.413
144.288
137.967
134.916
133.438
130.120
129.881
128.947
128.499
128.232
127.517
117.658
114.207
108.648
102.107
98.560
65.971
55.245
49.228
38.254
25.459

₩

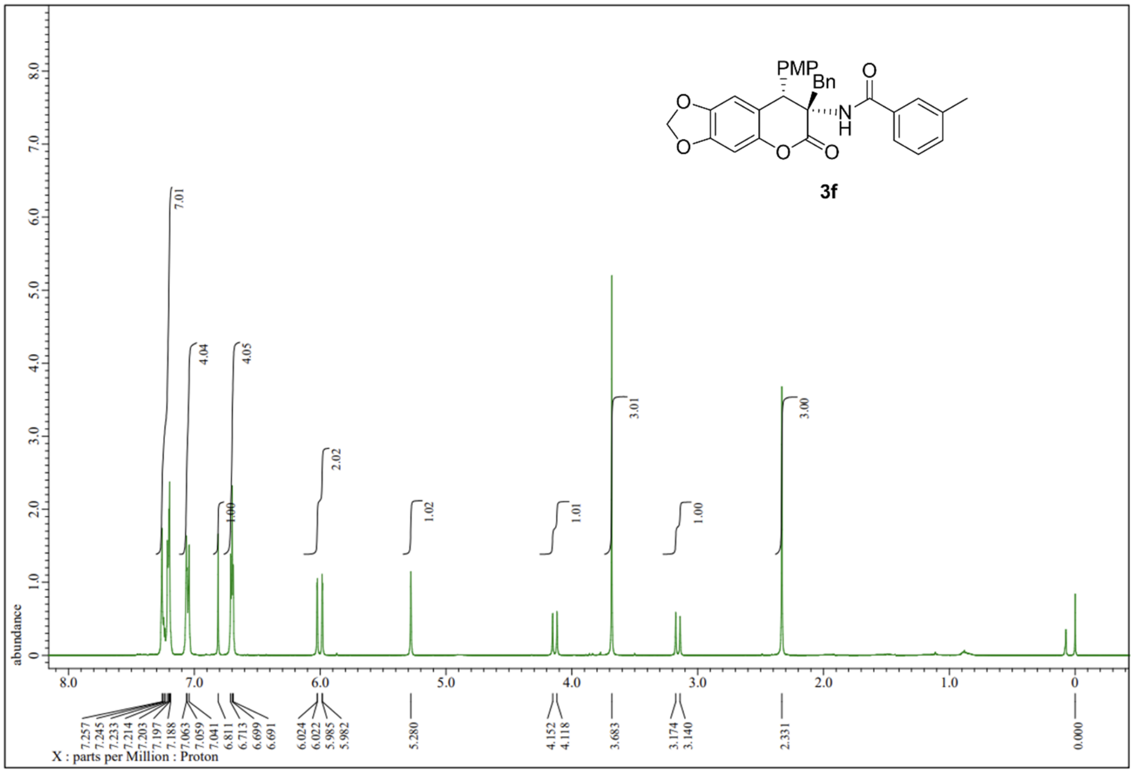
PMP
Bn
O
N
H
3f
abundance
X : parts per Million : Proton
7.01
4.04
4.05
1.00
2.02
1.02
1.01
3.01
1.00
3.00
7.257
7.245
7.233
7.214
7.203
7.197
7.188
7.063
7.059
7.041
6.811
6.713
6.699
6.691
6.024
6.022
5.985
5.982
5.280
4.152
4.118
3.683
3.174
3.140
2.331
0.000

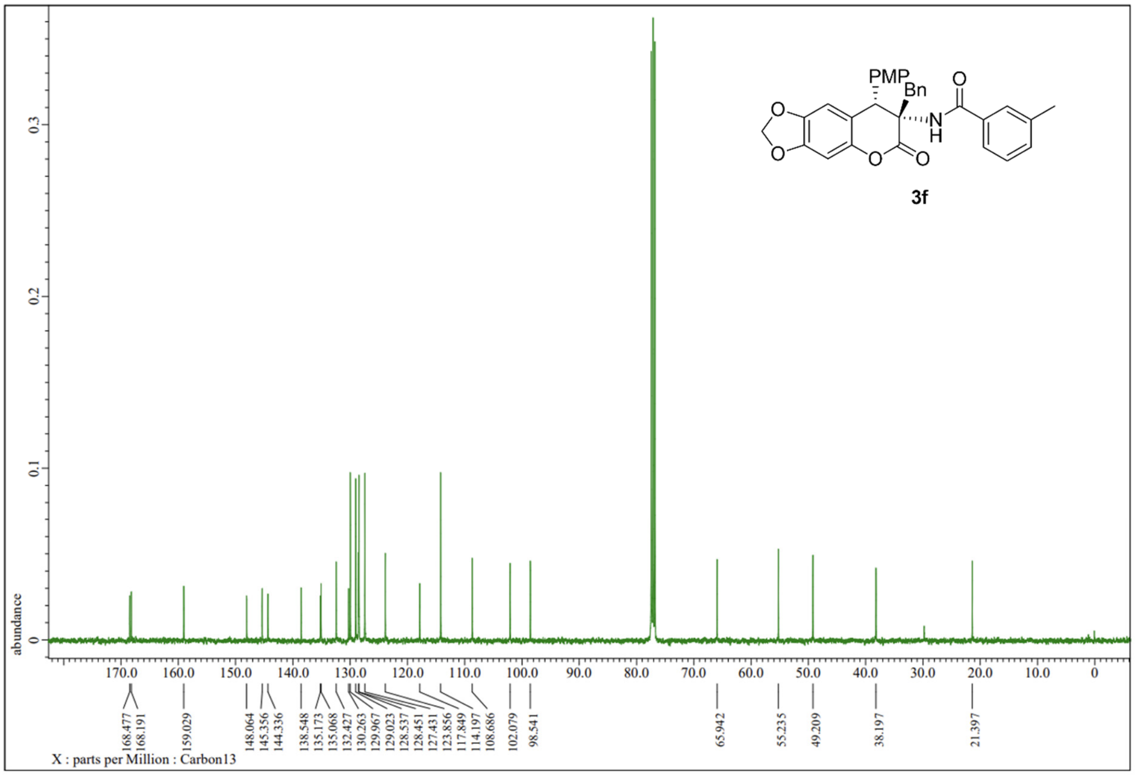
PMP
Bn
3f
abundance
168.477 168.191 159.029 148.064 145.356 144.336 138.548 135.173 135.068 132.427 130.263 129.967 129.023 128.537 128.451 127.431 123.856 117.849 114.197 108.686 102.079 98.541 65.942 55.235 49.209 38.197 21.397
X : parts per Million : Carbon13

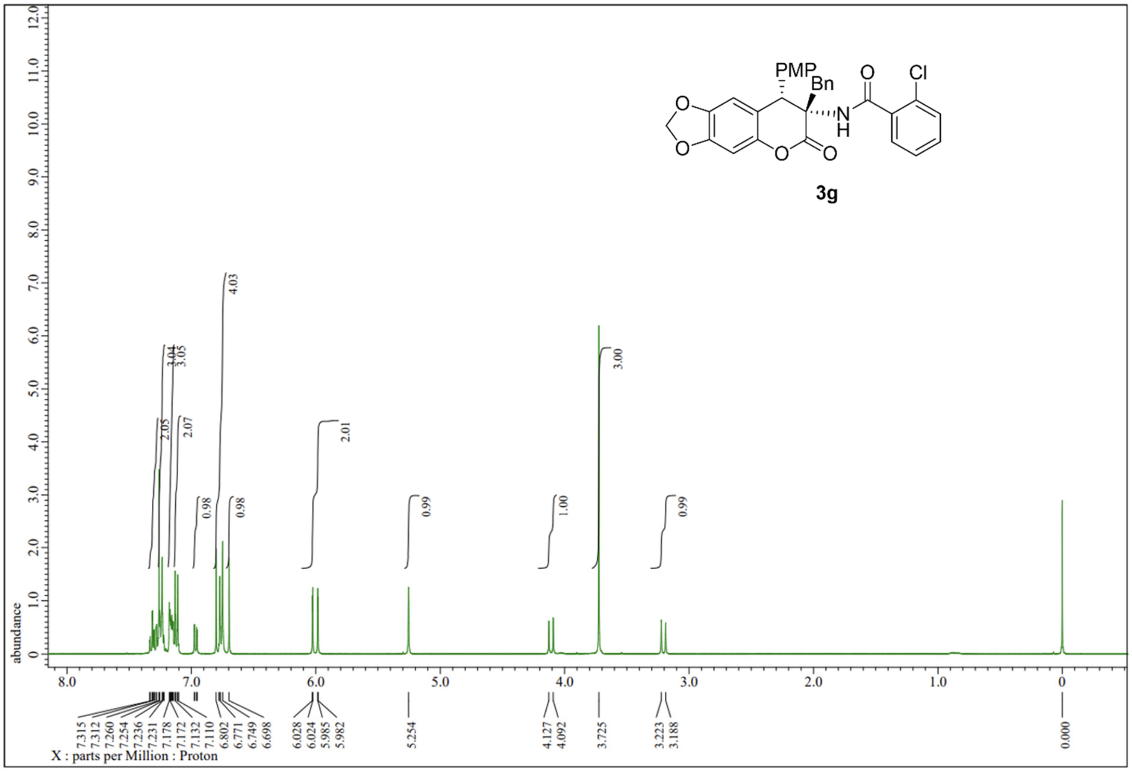
PMP
Bn
Cl
3g
abundance
2.05 3.04 3.05 2.07 0.98 4.03 0.98 2.01 0.99 1.00 3.00 0.99
7.315 7.312 7.260 7.254 7.236 7.231 7.178 7.172 7.132 7.110 6.802 6.771 6.749 6.698 6.028 6.024 5.985 5.982 5.254 4.127 4.092 3.725 3.223 3.188 0.000
X : parts per Million : Proton

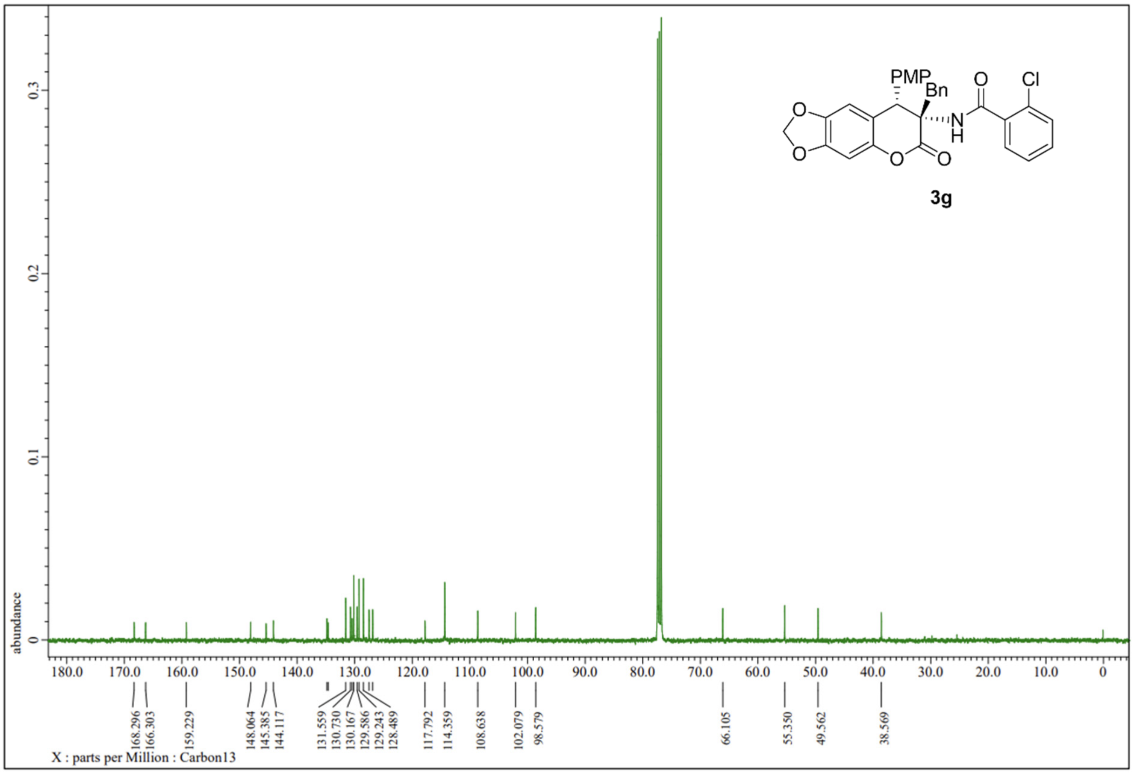
PMP
Bn
O
Cl
O
N
H
O
O
O
3g
abundance
0.3
0.2
0.1
0
180.0 170.0 160.0 150.0 140.0 130.0 120.0 110.0 100.0 90.0 80.0 70.0 60.0 50.0 40.0 30.0 20.0 10.0 0
168.296
166.303
159.229
148.064
145.385
144.117
131.559
130.730
130.167
129.586
129.243
128.489
117.792
114.359
108.638
102.079
98.579
66.105
55.350
49.562
38.569
X : parts per Million : Carbon13

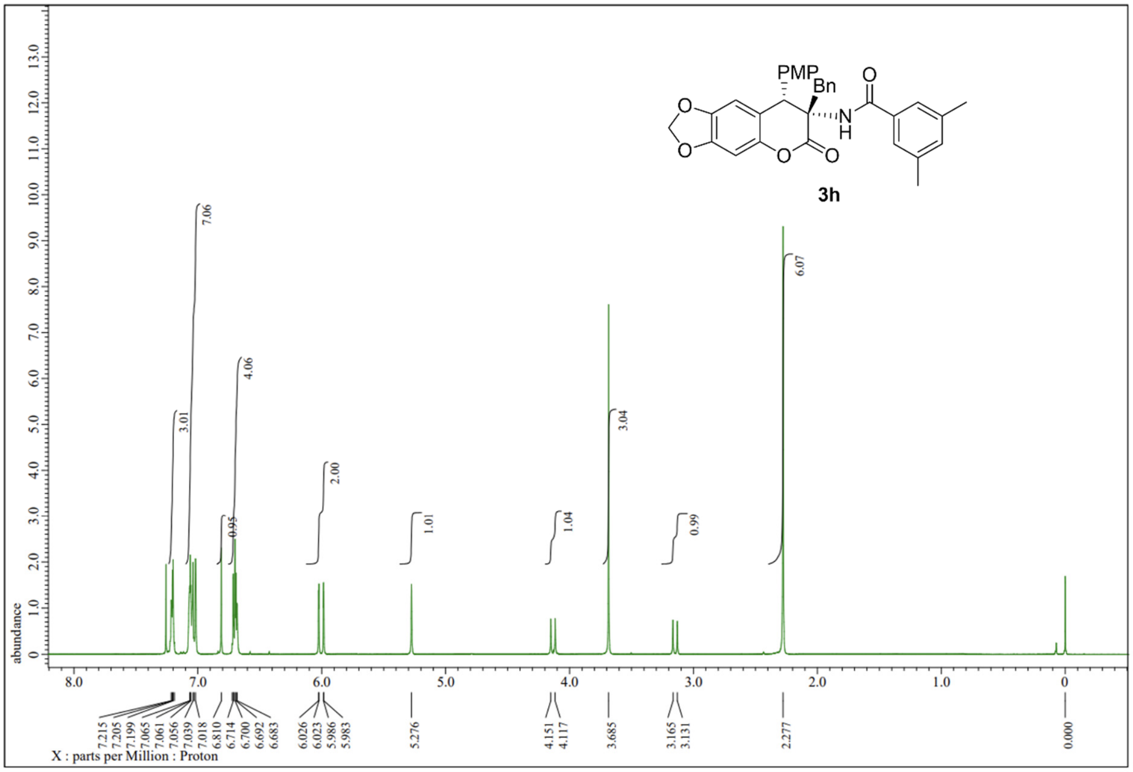
PMP
Bn
O
N
H
O
O
O
O
3h
abundance
13.0 12.0 11.0 10.0 9.0 8.0 7.0 6.0 5.0 4.0 3.0 2.0 1.0 0
3.01
7.06
0.95
4.06
2.00
1.01
1.04
3.04
0.99
6.07
8.0 7.0 6.0 5.0 4.0 3.0 2.0 1.0 0
7.215
7.205
7.199
7.065
7.061
7.056
7.039
7.018
6.810
6.714
6.700
6.692
6.683
6.026
6.023
5.986
5.983
5.276
4.151
4.117
3.685
3.165
3.131
2.277
0.000
X : parts per Million : Proton

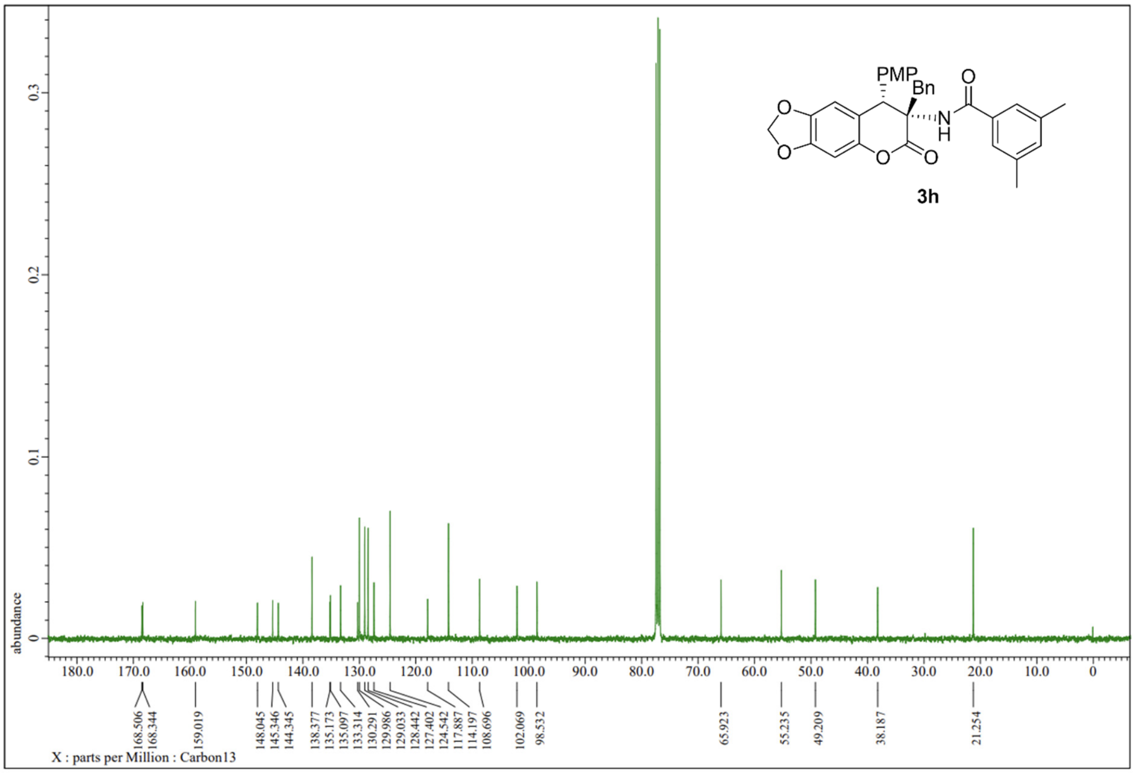
PMP
Bn
O
N
H
O
O
O
O
3h
abundance
0.3
0.2
0.1
0
180.0
170.0
160.0
150.0
140.0
130.0
120.0
110.0
100.0
90.0
80.0
70.0
60.0
50.0
40.0
30.0
20.0
10.0
0
168.506
168.344
159.019
148.045
145.346
144.345
138.377
135.173
135.097
133.314
130.291
129.986
129.033
128.442
127.402
124.542
117.887
114.197
108.696
102.069
98.532
65.923
55.235
49.209
38.187
21.254
X : parts per Million : Carbon13

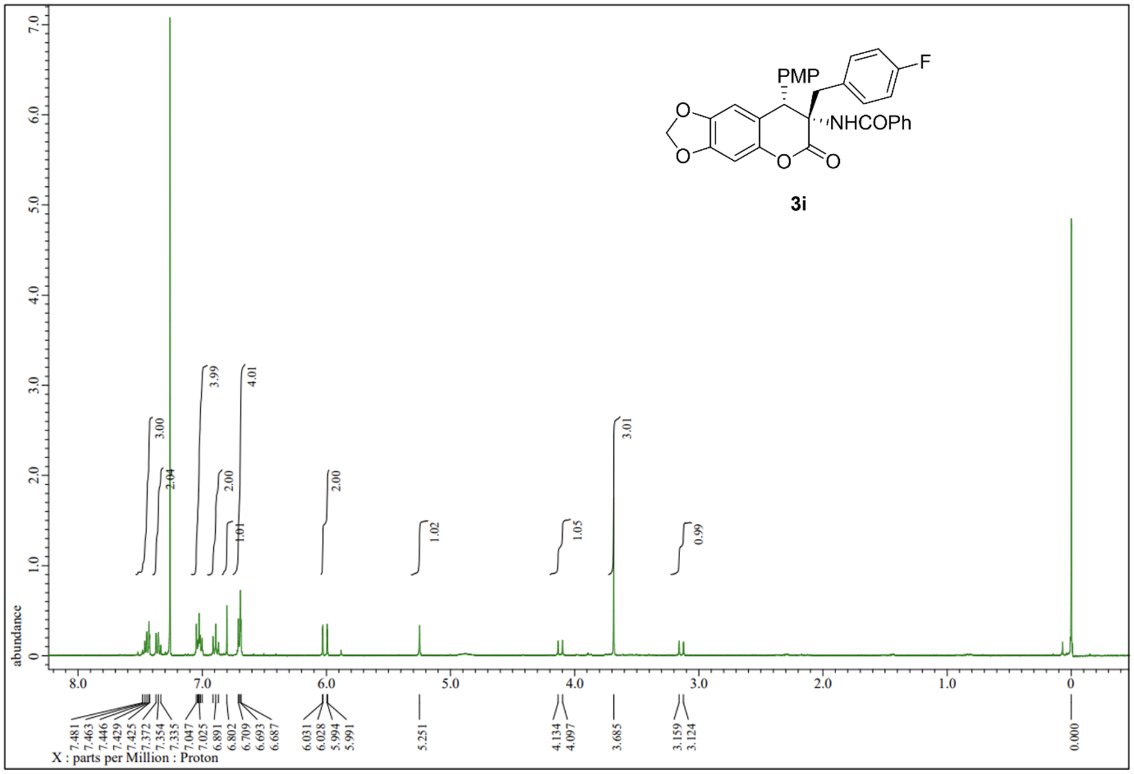
PMP
F
NHCOPh
O
O
O
O
3i
abundance
7.0
6.0
5.0
4.0
3.0
2.0
1.0
0
3.00
2.04
3.99
2.00
1.01
4.01
2.00
1.02
1.05
3.01
0.99
8.0
7.0
6.0
5.0
4.0
3.0
2.0
1.0
0
7.481
7.463
7.446
7.429
7.425
7.372
7.354
7.335
7.047
7.025
6.891
6.802
6.709
6.693
6.687
6.031
6.028
5.994
5.991
5.251
4.134
4.097
3.685
3.159
3.124
0.000
X : parts per Million : Proton

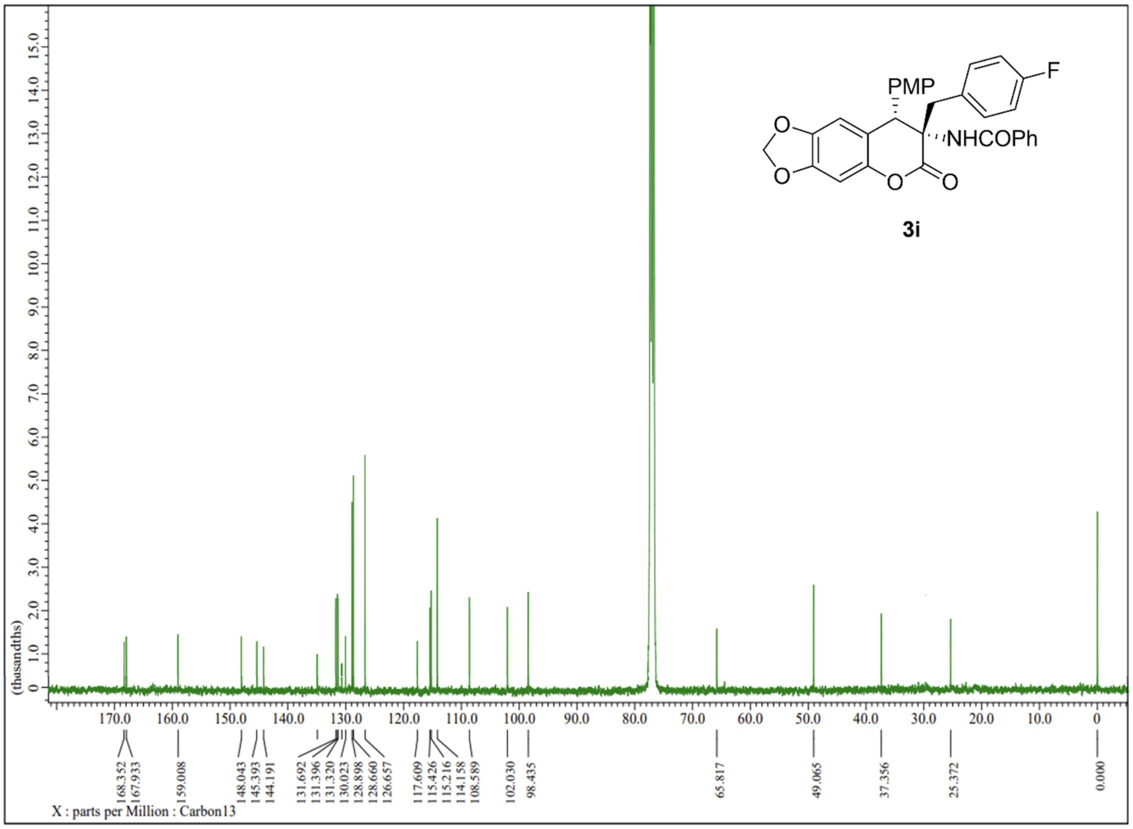
PMP
NHCOPh
F
O
O
O
O
3i
(thusandths)
X : parts per Million : Carbon13
168.352
167.933
159.008
148.043
145.393
144.191
131.692
131.396
131.320
130.023
128.898
128.660
126.657
117.609
115.426
115.216
114.158
108.589
102.030
98.435
65.817
49.065
37.356
25.372
0.000

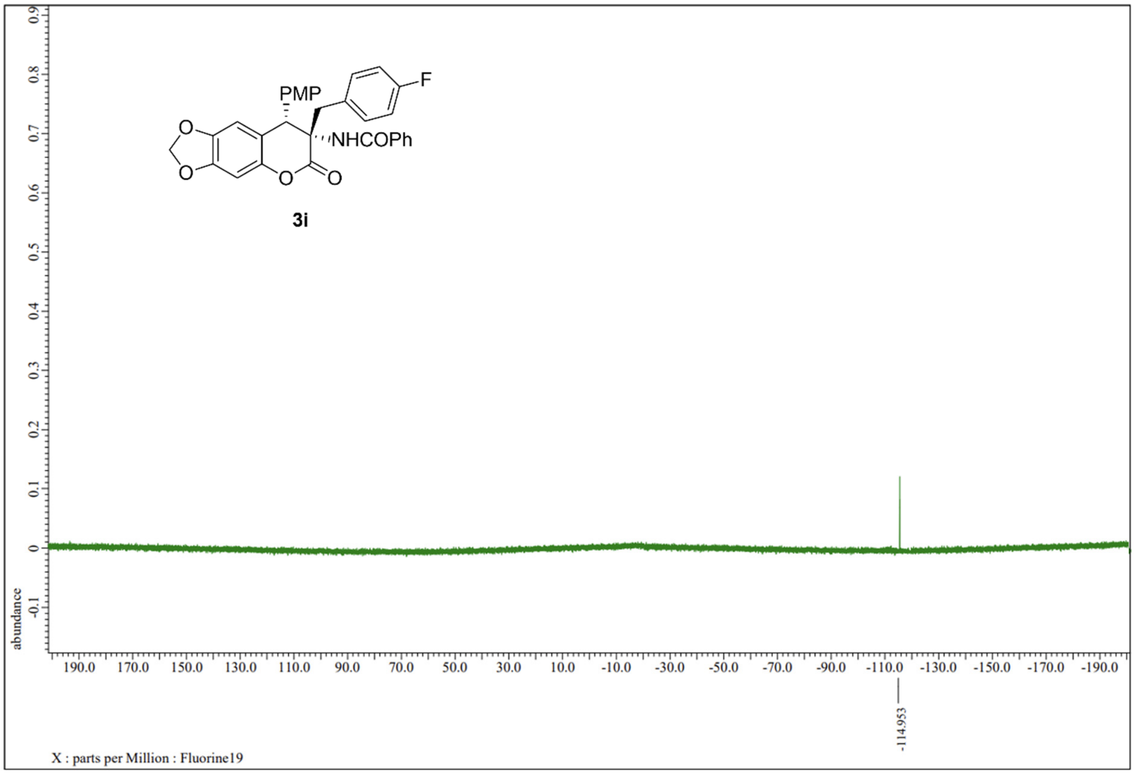
PMP
NHCOPh
F
O
O
O
O
3i
abundance
X : parts per Million : Fluorine19
-114.953

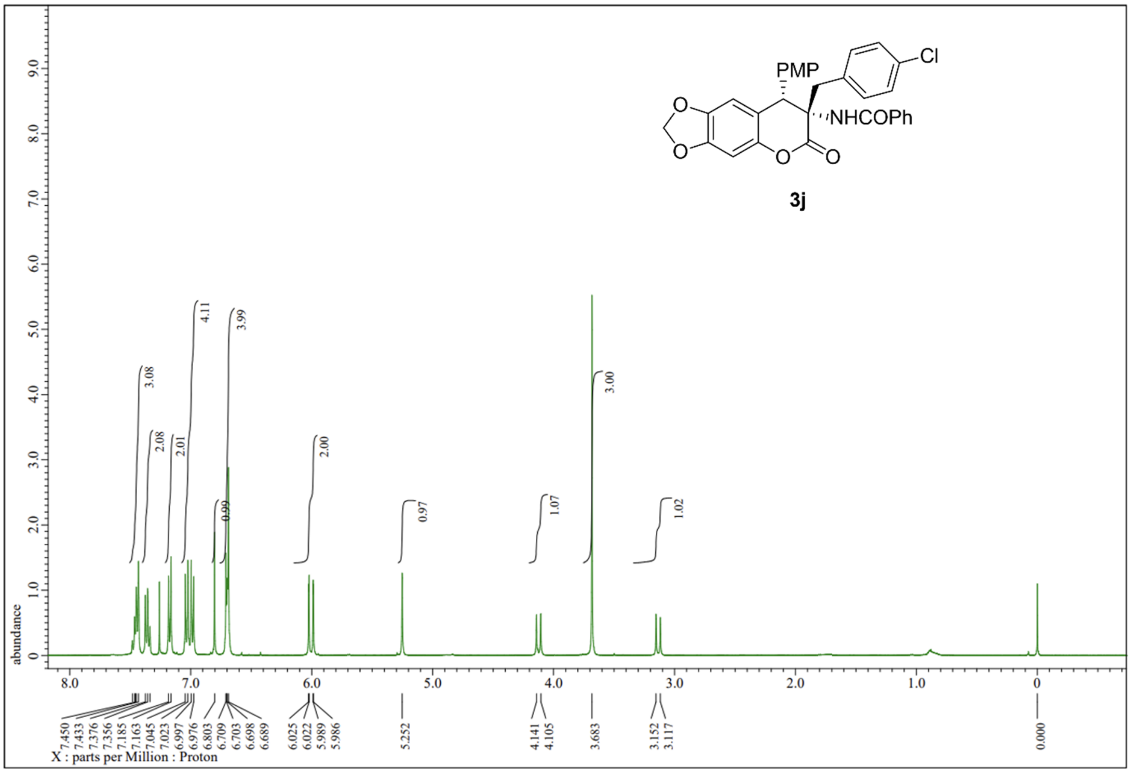
PMP
Cl
NHCOPh
3j
abundance
X : parts per Million : Proton
3.08
2.08
2.01
4.11
0.99
3.99
2.00
0.97
1.07
3.00
1.02
7.450
7.433
7.376
7.356
7.185
7.163
7.045
7.023
6.997
6.976
6.803
6.709
6.703
6.698
6.689
6.025
6.022
5.989
5.986
5.252
4.141
4.105
3.683
3.152
3.117
0.000

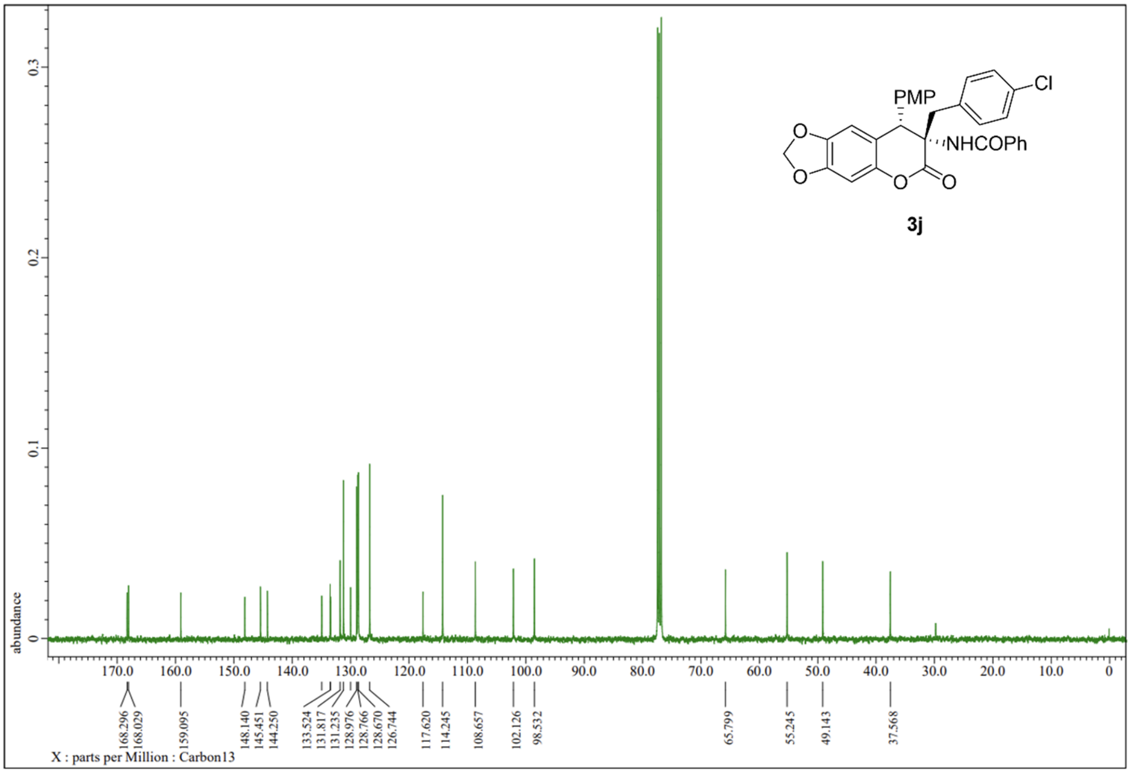
PMP
Cl
NHCOPh
3j
abundance
X : parts per Million : Carbon13
168.296
168.029
159.095
148.140
145.451
144.250
133.524
131.817
131.235
128.976
128.766
128.670
126.744
117.620
114.245
108.657
102.126
98.532
65.799
55.245
49.143
37.568

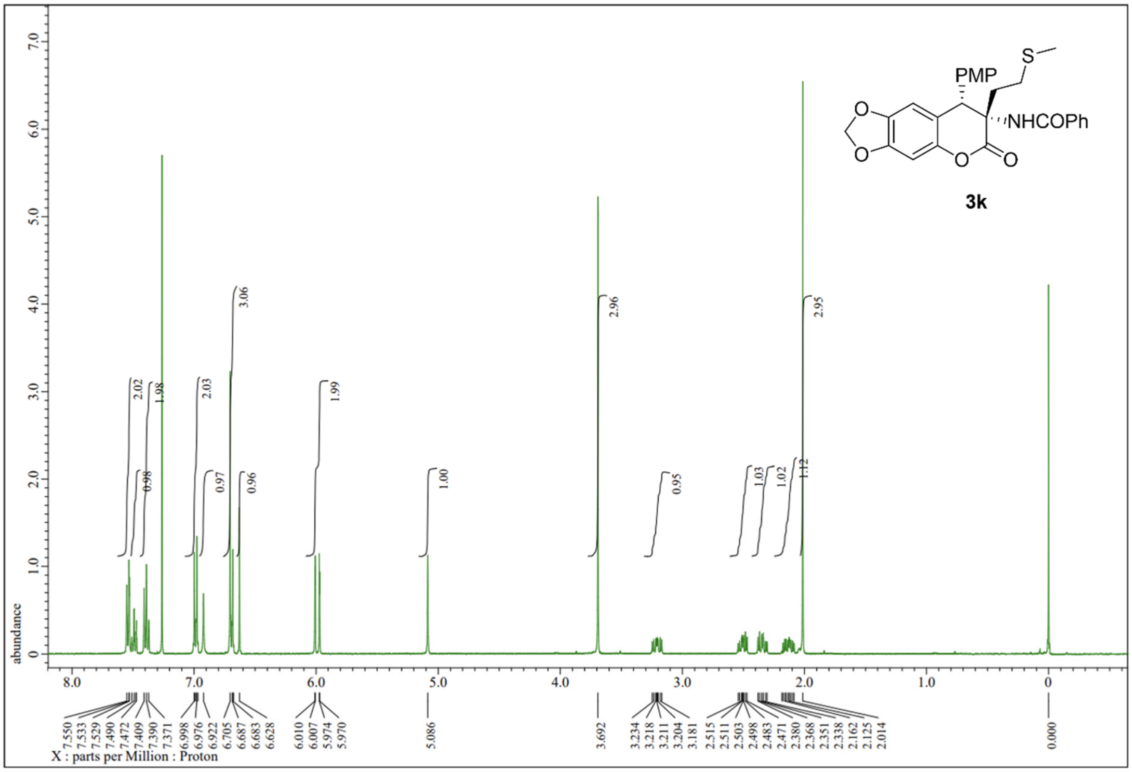
PMP
NHCOPh
3k
abundance
X : parts per Million : Proton

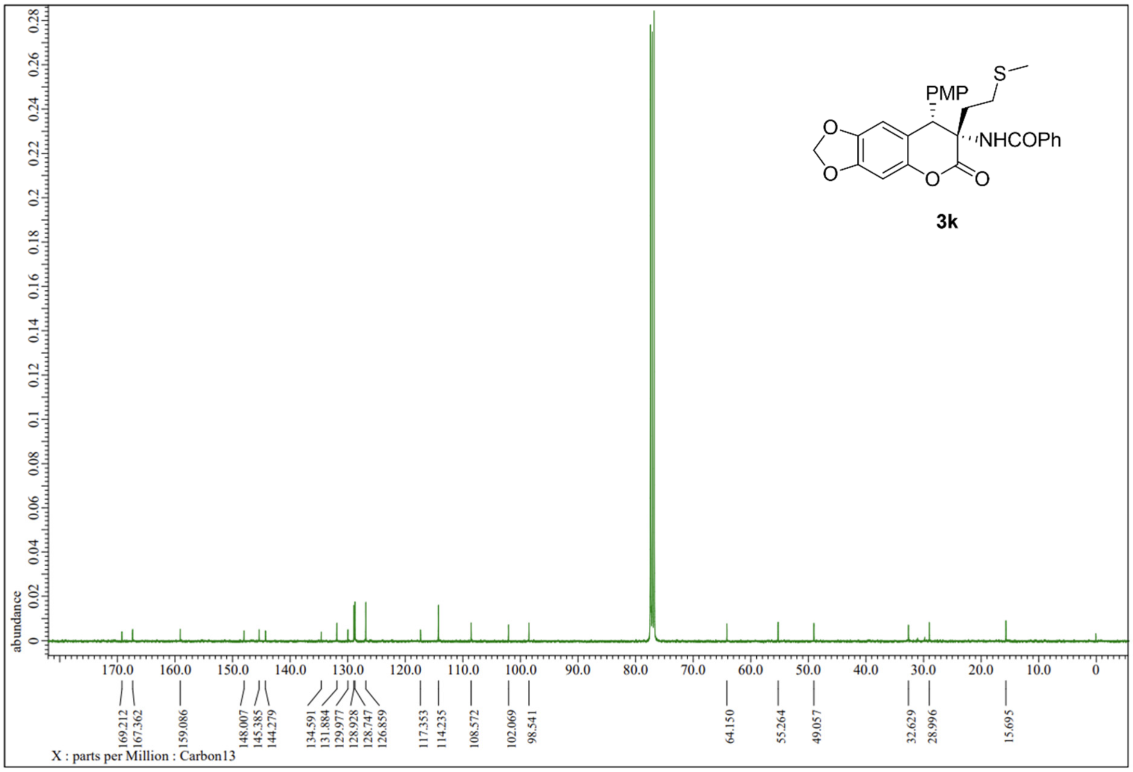
PMP
NHCOPh
3k
abundance
X : parts per Million : Carbon13

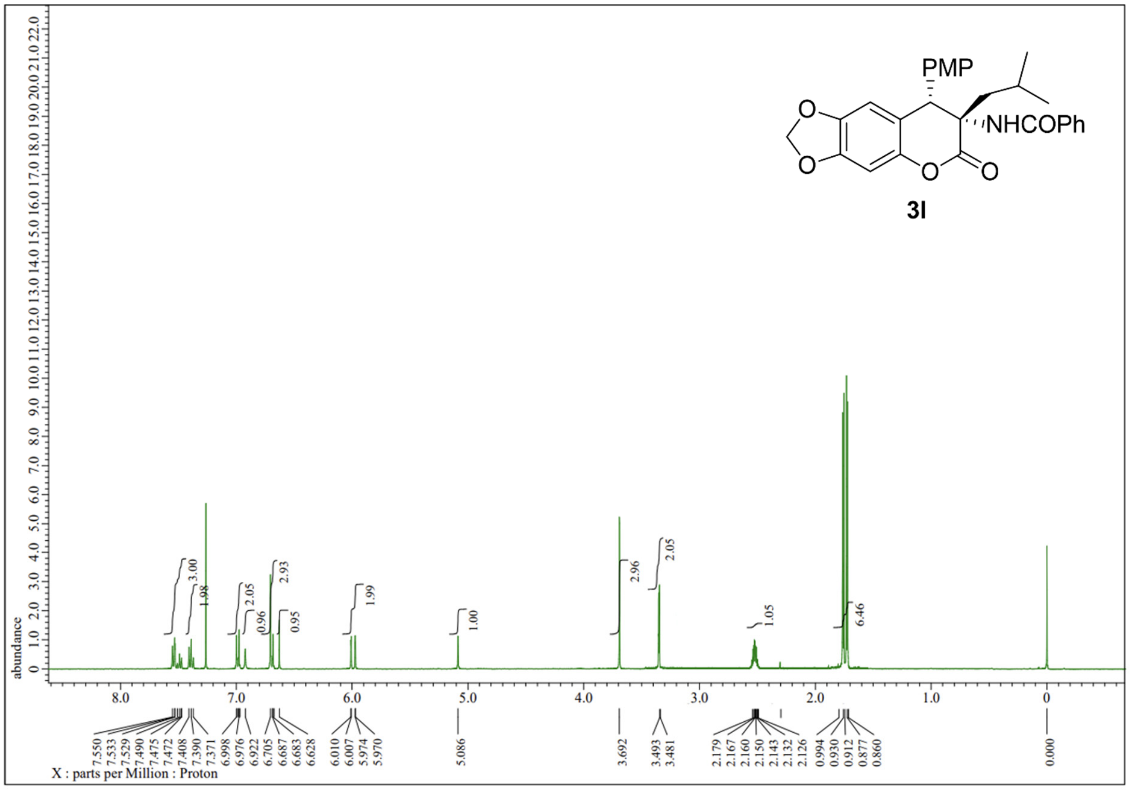
PMP
NHCOPh
3l
abundance
X : parts per Million : Proton

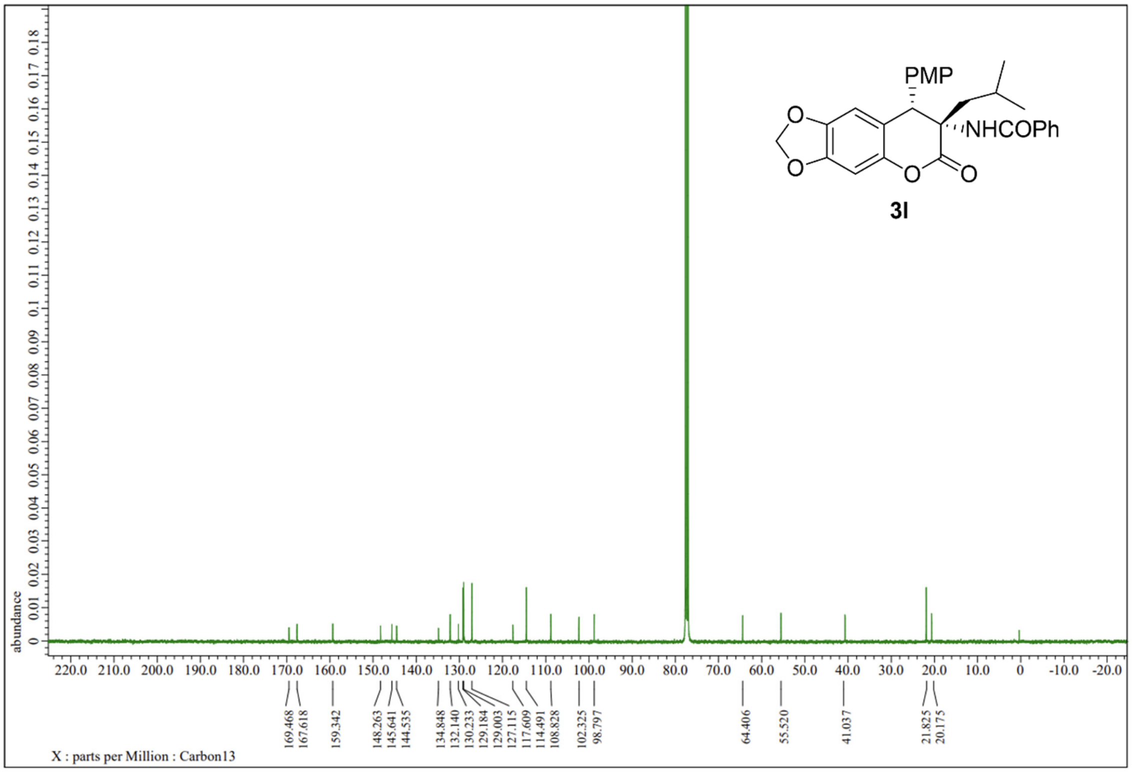
PMP
NHCOPh
3l
abundance
X : parts per Million : Carbon13

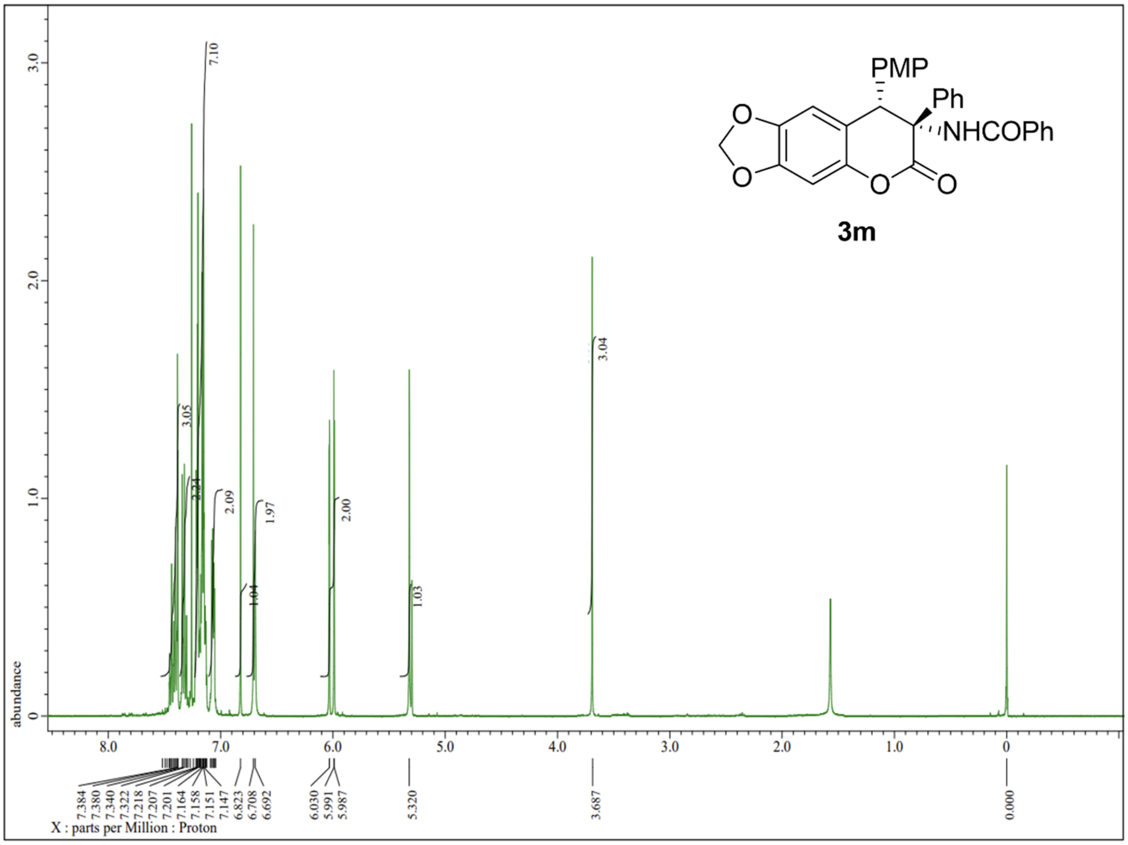
PMP
Ph
NHCOPh
3m
abundance
X : parts per Million : Proton

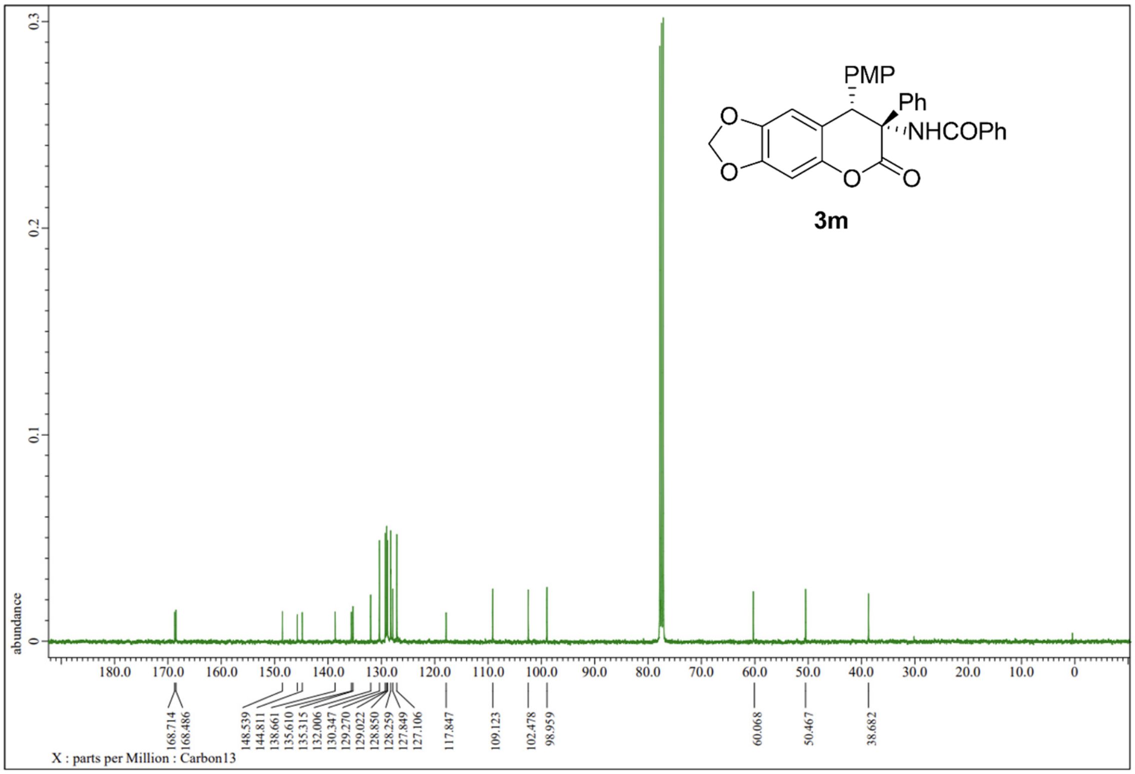
PMP
Ph
NHCOPh
3m
abundance
X : parts per Million : Carbon13

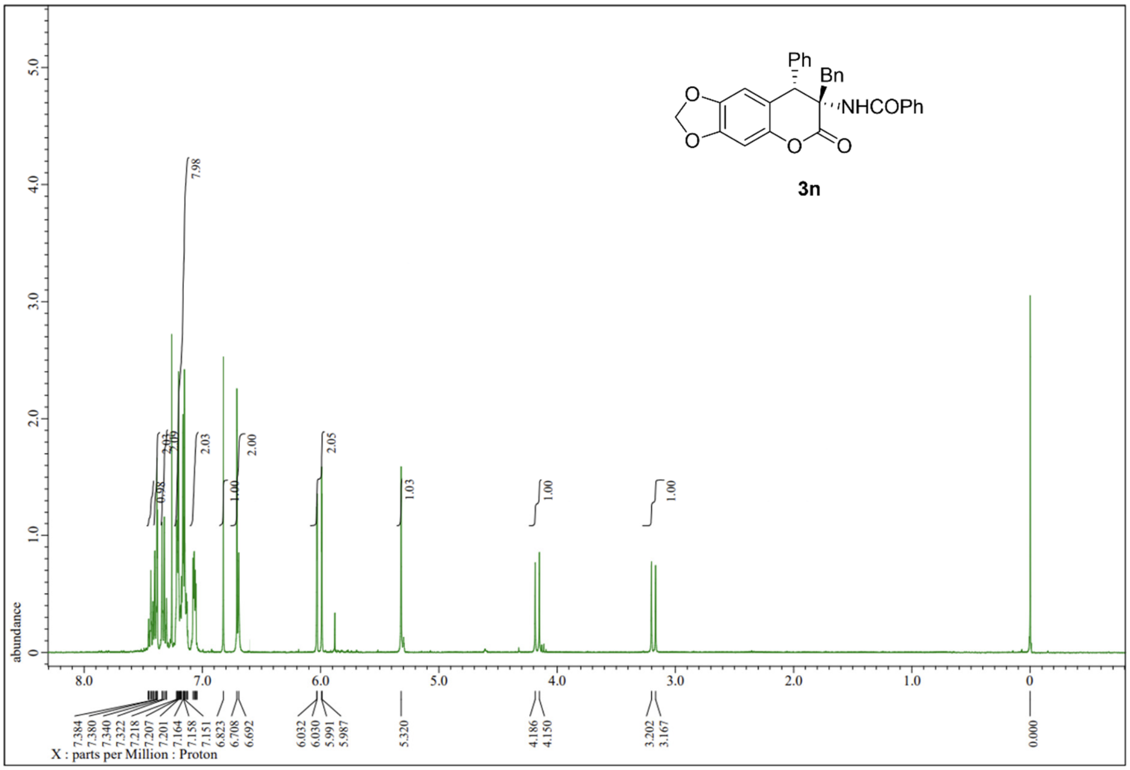
Ph
Bn
NHCOPh
O
O
O
O
3n
abundance
X : parts per Million : Proton
7.98
0.98
2.03
2.00
2.03
1.00
2.00
2.05
1.03
1.00
1.00
7.384 7.380 7.340 7.322 7.218 7.207 7.201 7.164 7.158 7.151 6.823 6.708 6.692 6.032 6.030 5.991 5.987 5.320 4.186 4.150 3.202 3.167 0.000

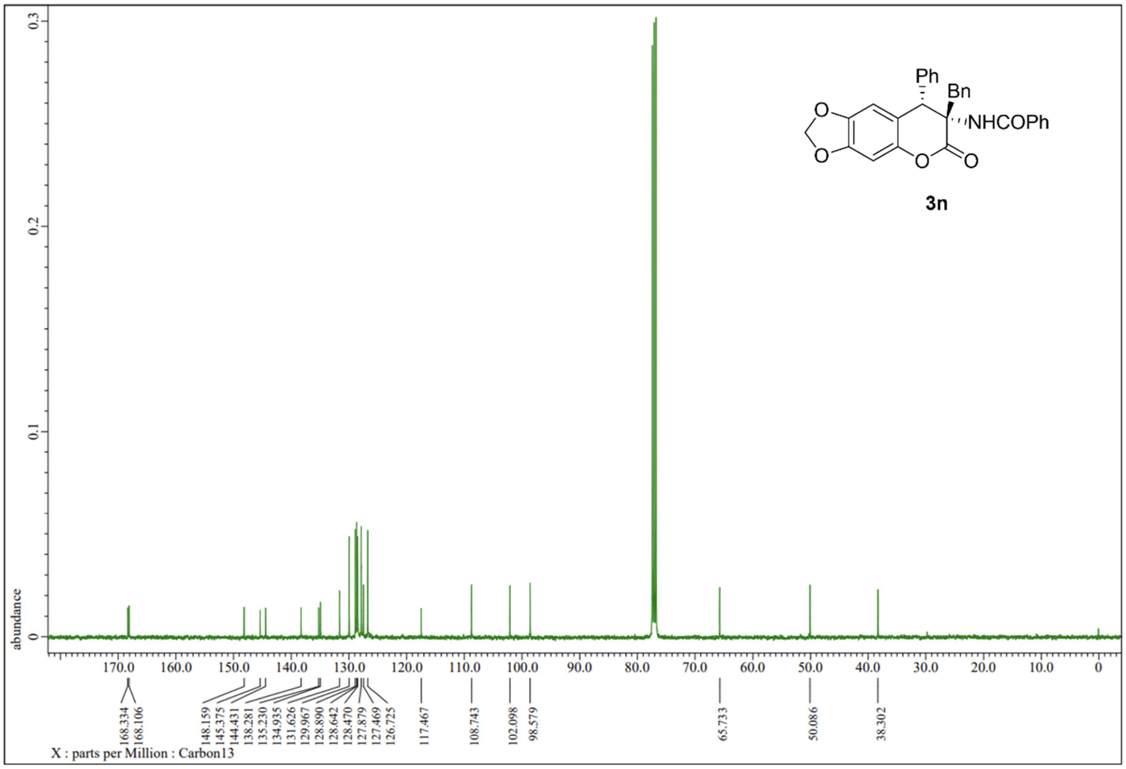
Ph
Bn
NHCOPh
O
O
O
O
3n
abundance
X : parts per Million : Carbon13
168.334 168.106 148.159 145.375 144.431 138.281 135.230 134.935 131.626 129.967 128.890 128.642 128.470 127.879 127.469 126.725 117.467 108.743 102.098 98.579 65.733 50.086 38.302

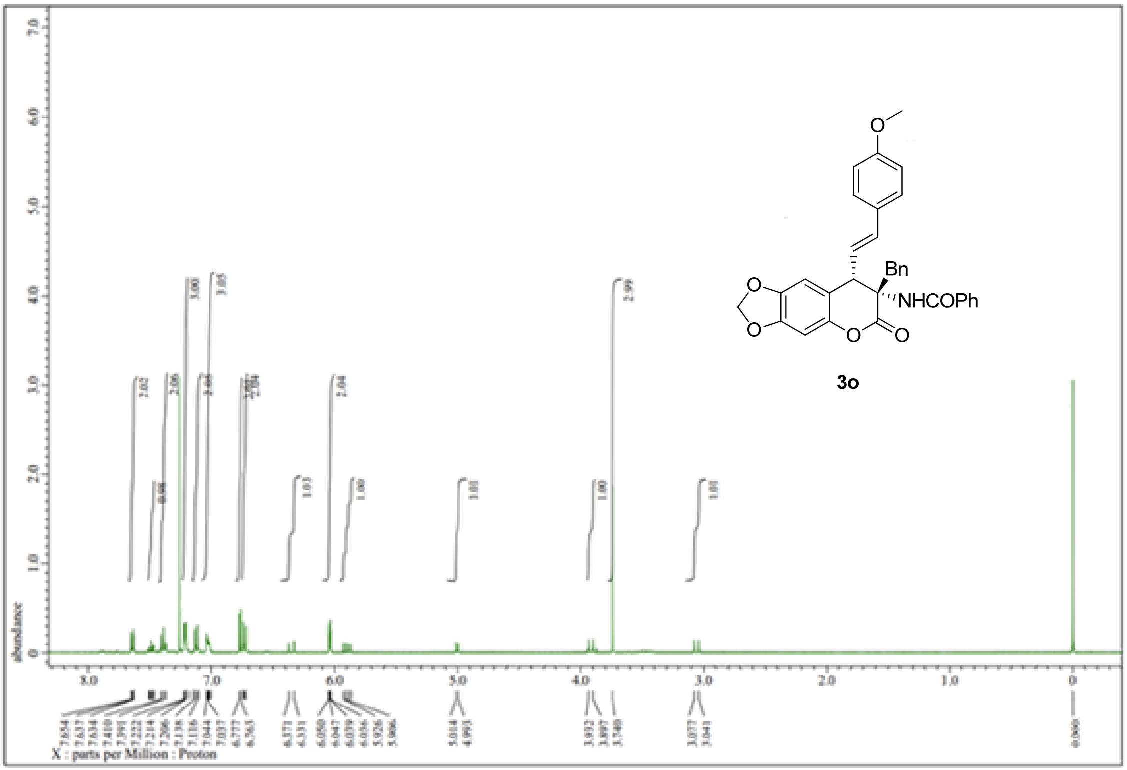
abundance
Bn
NHCOPh
3o
X : parts per Million : Proton

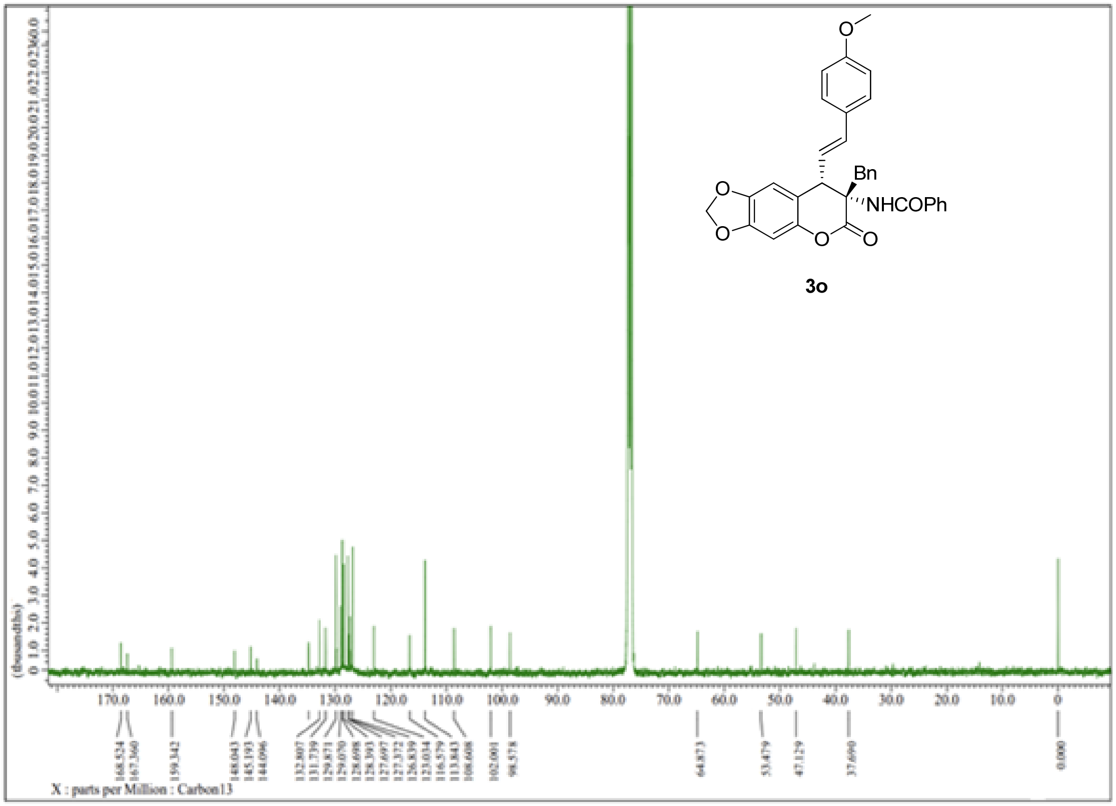
(thousandths)
Bn
NHCOPh
3o
X : parts per Million : Carbon13
